# Calcium Dobesilate Restores Autophagy by Inhibiting the VEGF/PI3K/AKT/mTOR Signaling Pathway

**DOI:** 10.3389/fphar.2019.00886

**Published:** 2019-08-09

**Authors:** Yue Wang, Yun-hong Lu, Chao Tang, Mei Xue, Xiao-yu Li, Yun-peng Chang, Ying Cheng, Ting Li, Xiao-chen Yu, Bei Sun, Chun-jun Li, Li-ming Chen

**Affiliations:** NHC Key Laboratory of Hormones and Development (Tianjin Medical University), Tianjin Key Laboratory of Metabolic Diseases, Tianjin Medical University Chu Hsien-I Memorial Hospital, Tianjin Institute of Endocrinology, Tianjin, China

**Keywords:** diabetic kidney disease, calcium dobesilate, autophagy, albuminuria, VEGF, VEGFR2

## Abstract

**Objective:** Calcium dobesilate (CaD), an effective drug for the treatment of diabetic microvascular complications, especially diabetic retinopathy, is widely used in the clinic. Interestingly, several studies have indicated that CaD is therapeutic for diabetic kidney disease (DKD). Recently, evidence has indicated that altered vascular endothelial growth factor (VEGF) expression and decreased autophagy are the main pathological mechanisms of proteinuria. Thus, this study was conducted to explore the effect of CaD on restoring autophagy in DKD and the possible signaling pathway between VEGF and autophagy.

**Methods:** Obese mice with spontaneous diabetes (KK-Ay) and high-fat diet- and streptozotocin-induced diabetic mice (HFD/STZ) were used in this study. Biochemical staining, western blotting, and immunohistochemistry were conducted to determine the angioprotective effect of CaD and the underlying mechanism between autophagy and VEGF/VEGFR.

**Results:** Our results showed that CaD was capable of reducing albuminuria and restoring renal histological changes in KK-Ay and HFD/STZ-induced diabetic mice. CaD restored autophagy by decreasing the protein expression of LC3 II, Atg5, and beclin 1 and increasing the expression of P62. Moreover, CaD reduced the activation of the autophagy-related PI3K/AKT/mTOR pathway possibly *via* decreasing VEGF and downregulating VEGF receptor 2.

**Conclusion:** Overall, CaD, as a novel potential therapeutic drug for DKD, plays a key role in protecting renal function and restoring autophagy by blocking VEGF/VEGFR2 and inhibiting the PI3K/AKT/mTOR signaling pathway.

## Introduction

Diabetic kidney disease (DKD) is a major microvascular complication of diabetes mellitus and one of the leading factors of morbidity and mortality in diabetic patients (Semenkovich et al., 2015). According to the latest statistical data, DKD is responsible for 40–50% of all cases of end-stage renal disease (ESRD) (Collins et al., 2012). To date, there is no curative therapeutic strategy for DKD; therefore, it is urgent to explore the underlying mechanism and develop specific and effective therapies. Currently, the major method for improving prognosis and alleviating symptoms is decreasing proteinuria, which is a major characteristic of DKD and the progression to ESRD, and improving renal function, which is reflected by decreased estimated glomerular filtration rate (eGFR) (Yoon et al., 2004). One of the leading factors that accelerates the production of proteinuria is increased glomerular filtration membrane permeability for albumin and other proteins. The persistent dysfunction of the glomerular filtration barrier (GFB) is responsible for the progressive albuminuria of DKD patients under hyperglycemia conditions. Therefore, maintaining the structure and function of the microvasculature to reduce proteinuria in the early stage of DKD is indispensable for protecting renal function.

Autophagy is a key process in eukaryotic cellular recycling, and it addresses breakdown products and futile materials for cellular homeostasis under various stress conditions. This process is governed by upstream active factors, such as mTORC1, AMPK, ULK1, and mTORC1 (Dunlop and Tee, 2014). In recent years, it has been uncovered that altered autophagy in vascular endothelial cells, epithelial cells, pericytes, podocytes, and mesangial cells leads to imbalanced homeostasis and abnormal hemodynamics (Lenoir et al., 2015). A study showed that podocyte injury and severe proteinuria occur in diabetic mice with podocyte-specific autophagy deficiency and damaged lysosomes (Yasuda-Yamahara et al., 2015). It has been revealed that the pharmacological induction of autophagy has promising therapeutic potential to improve kidney function in diabetic patients (Lenoir et al., 2015).

Calcium dobesilate (calcium dihydroxy-2,5-benzenesulfonate, CaD), an artificially synthesized small molecule with antioxidative, anti-inflammatory, and anti-angiogenic properties, is now widely applied to the treatment of chronic vascular diseases, especially diabetic retinopathy. However, the specific mechanism is not yet clear. In addition, a number of studies have shown that CaD is a promising therapeutic strategy for microvascular disease not only in the retina but also in the kidney (Yoon et al., 2004). Moreover, CaD has been confirmed to be capable of restoring altered vascular permeability under hyperglycemic conditions (Angulo et al., 2011). It is known that the abnormal expression of vascular endothelial growth factor (VEGF) is closely related to increased vascular permeability. At present, VEGF is a therapeutic target for maintaining renal functionality (Advani et al., 2007). VEGF is primarily produced by podocytes and acts by binding to its receptor (Ferrara et al., 2003). It is clear that VEGF initiates and maintains the neovascularization process, which is injurious to the glomerular basement membrane (GBM) (Cooper et al., 1999). CaD plays a key role in inhibiting VEGF not only by interfering with its receptor binding but also by regulating VEGF activity by specifically interfering with its interaction with glycoproteins (Haller et al., 2017). Lower levels of receptor binding lead to lower levels of downstream signal activation. Therefore, vascular permeability is restored. Abnormal autophagy and VEGF expression can lead to altered permeability, increased urinary albuminuria excretion, and abnormal renal function.

In the present study, our objective was to explore the underlying mechanism by which CaD restores autophagy in the kidneys of DKD mice. We also aimed to reveal the possible signaling pathway between VEGF and autophagy. We found that CaD has a significantly protective effect on reducing vascular permeability and decreasing proteinuria. CaD is a promising and prospective treatment for DKD and other microvascular diseases.

## Materials and Methods

### Experimental Animals

Two types of rodent models were used in our experiments: obese mice with spontaneous diabetes (KK-Ay) (Risa et al., 2017; Yin et al., 2017) and high-fat diet- and streptozotocin-induced diabetic mice (HFD/STZ). 6-week-old male KK-Ay mice and C57BL/6 mice (25 ± 1 g) were obtained from Beijing HFK Bioscience Co., Ltd. (Beijing, China), and all mice were housed at 24 ± 2°C. All mice were given free access to chow and water. The study was approved by the ethical committee of Tianjin Medical University, and approval for the animal study was acquired from the Guide for the Care and Use of Laboratory Animals of the National Institutes of Health as well as the guidelines of the Animal Welfare Act.

### Experimental Procedures

KK-Ay mice were fed an HFD for 14 days. Blood glucose and body weight increased significantly. Then, KK-Ay mice were randomly divided into two groups: the diabetic control group (KK-Ay, *n* = 15) and the CaD-treated group (KK-Ay+CaD, *n* = 15). As age-matched male C57BL/6 mice are commonly used as nondiabetic controls for KK-Ay mice, we randomly selected C57BL/6 mice (25 ± 1 g) as the blank control group (NK, normal control, *n* = 15). The mice in the KK-Ay+CaD group were treated intragastrically with CaD at a dose of 200 mg/(kg·d) (McMahon et al., 1994; Villegas et al., 2005; Yang et al., 2013) daily for 12 weeks. The CaD powder was dissolved in water at a concentration of 10%. The CaD powder was purchased from Ebewe Pharma Ges.m.b.H.Nfg.KG (A-4866 Unterach, Austria).

Meanwhile, 55 C57BL/6 mice (19 ± 1 g) were randomly divided into two groups: the standard diet group (NC, normal control, *n* = 15) and the HFD group (*n* = 40). The HFD consisted of 48.5% carbohydrate, 17.5% protein, and 17.9% fat. It was purchased from HFK Bio-Technology Co., Ltd. (Beijing, China). After 12 weeks, blood glucose levels and insulin resistance in the HFD group were measured by the intraperitoneal glucose tolerance test (IPGTT). Then, HFD mice were intraperitoneally injected with streptozotocin (STZ) at a dose of 30 mg/(kg·d) for seven consecutive days (Breyer et al., 2005). Then, blood glucose levels were tested within 72 h. The successful modeling rate was not ideal. Thus, we continued the injection at the same dose for three more days. After 10 days of injection, the blood glucose of 30 mice exceeded 16.6 mmol/L (Xiao et al., 2018), and those 30 mice were considered qualified models of diabetes. The 10 unsuccessful model mice were excluded. The 30 HFD/STZ-induced diabetic mice were randomly divided into two groups: the diabetic control group (HFD/STZ, *n* = 15) and the CaD treatment group (HFD/STZ+CaD, *n* = 15). The HFD/STZ+CaD group received CaD intragastrically at a dose of 200 mg/(kg·d) for 12 weeks. The control groups received vehicle at the same dose for the same amount of time.

### Intraperitoneal Glucose Tolerance Test (IPGTT)

After being fed an HFD for 12 weeks, the HFD mice were fasted overnight for 12 h and peritoneally injected with glucose at a dose of 2 g/kg (Dusane and Joshi, 2013). The caudal vein blood glucose level was measured 0, 15, 30, 60, 90, and 120 min after the glucose load.

### Biochemical Analysis

After 12 weeks of treatment with CaD or vehicle, the mice were sacrificed under anesthesia. Blood samples were collected from the retroorbital venous plexus, and urine samples were collected from specific metabolic cages. The blood and urine samples were analyzed for fasting blood glucose (FBG), urinary albumin/creatinine (ACR), total triglyceride (TG), total cholesterol (TC), alanine transaminase (ALT), aspartate transaminase (AST), and serum creatinine (Scr) levels with an automatic biochemistry analyzer (Roche). Based on FBG, UAE, and ACR levels, all mice were diagnosed with DKD.

### Histopathological Examination

Kidney samples were immersed in 4% paraformaldehyde and embedded in paraffin, and cross-sections were prepared for hematoxylin/eosin (HE) staining, Masson staining, and periodic acid-Schiff (PAS) staining (Leagene Biotechnology, Beijing, China). 20–30 glomeruli in each animal were measured. The mean glomerular volume (MGV) was determined from the mean glomerular cross-sectional area (MGA) and calculated as:

$$\mathrm{MGV}=\beta /\text{K x }{\left(\mathrm{MGA}\right)}^{3/2}\text{,}$$

where ß = 1.38 is the shape coefficient for a sphere and *K* = 1.1 is the distribution coefficient (Malatiali et al., 2016). The mesangial matrix area (MMA) was defined as PAS-positive mesangial area in each glomerular tuft (Malatiali et al., 2008). Examination of the sections was performed under a microscope at a magnification of 400×, and all images were quantified with Image-Pro Plus 6.0 software.

### Immunohistochemistry

The kidneys were immersed in 4% paraformaldehyde and embedded in paraffin. Kidney cross-sections were dewaxed heated for antigen retrieval, incubated with 3% H_2_O_2_ to block endogenous peroxidase activity and blocked with 1% BSA. Then, the sections were incubated at 4°C overnight with the following primary antibodies: anti-LC3 (B) (Cell Signaling Technology, 1:50), anti-P62/SQSTM1 (Abcam, 1:100), anti-nephrin (Abcam, 1:100), and anti-podocin (Abcam, 1:100). The sections were incubated with the relevant secondary antibodies, and the positive staining was visualized by staining with diaminobenzidine (DAB) and counterstaining with hematoxylin and examined under an electron microscope at a magnification of 400×. All images were quantified with Image-Pro Plus 6.0 software.

### Western Blotting Analysis

Kidney lysates were prepared with RIPA lysis buffer containing phenylmethanesulfonyl fluoride (PMSF) and phosphate inhibitor (100:1:1). Equal amounts of selected protein extract samples were loaded onto sodium dodecyl sulfate - polyacrylamide gel electrophoresis (SDS-PAGE) gels and transferred to polyvinylidene difluoride (PVDF) membranes (0.45 μm and 0.22 μm, Millipore, MA, USA). After being blocked for 2 h in 5% skim milk/TBST, the PVDF membranes were washed with Tris-buffered saline containing 0.1% Tween-20 (TBST) three times and incubated overnight at 4°C with different primary antibodies (anti-PI3K, 1:1,000; anti-p-PI3K, 1:1,000; anti-Akt, 1:1,000; anti-p-Akt, 1:1,000; anti-mTOR, 1:1,000; anti-p-mTOR, 1:1,000; anti-LC3, 1:500; anti-p62/SQSTM1, 1:1,000. anti-ATG5, 1:1,000; anti-beclin 1, 1:1,000; anti-VEGF, 1:1,000; anti-VEGFR2, 1:1,000; and anti-β-tubulin 1:2,000). The abovementioned primary antibodies were all obtained from Cell Signaling Technology (Danvers, MA, USA). Next, the PVDF membranes were washed with TBST three times and incubated with horseradish peroxidase (HRP)-conjugated secondary antibodies (Cell Signaling Technology, Danvers, MA, USA) at room temperature for approximately 1 h. The immunoreactive of the bands was visualized by Bio-Rad Image Lab with an electrochemiluminescence system (ECL). The densitometric analysis of the protein bands was accomplished by ImageJ (NIH image software) and was normalized to relevant controls.

### Statistical Analysis

The data are presented as the mean ± standard error of the mean (SEM), and the statistical analysis was carried out by one-way analysis of variance (ANOVA) for comparisons of more than three groups. A *P* value of 0.05 or less was considered significant. Graphical results were analyzed using GraphPad Prism 8 (GraphPad Software, Inc., La Jolla, CA, USA). Statistical and data analyses were performed with IBM SPSS software 20.0 (SPSS, Chicago, IL, USA).

## Results

### Calcium Dobesilate Decreased Albuminuria in KK-Ay and HFD/STZ Mice

**Figure 1:**
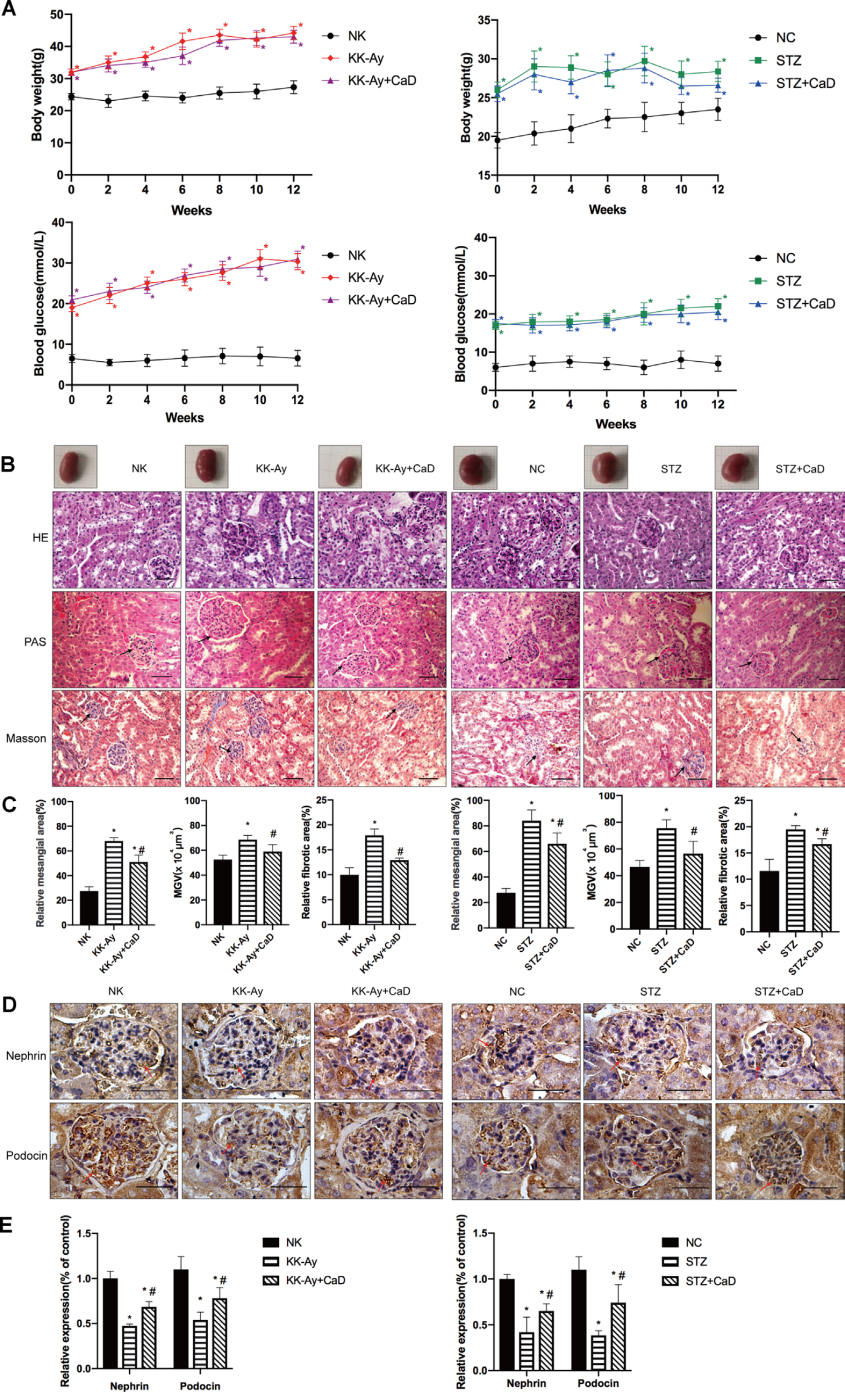
Calcium dobesilate (CaD) restored hyperglycemia-induced renal histological changes. **(A)** Body weight and blood glucose measured after 12 weeks of CaD treatment. **(B)** The measurement of kidney samples of the mice compared to unified size markers. HE, PAS, and Masson staining of kidney samples. **(C)** The results of MMA, MGV, and Masson staining analysis. **(D, E)** The immunohistochemical quantification and analysis of nephrin and podocin in kidney samples. Original magnification of 400×. The scale bars in the right lower corner represent 100 µm. The error bars represent the SEM. The arrows indicate deposited glomerular mesangial matrix in the PAS-stained tissue, glomerular and tubulointerstitial fibrosis in Masson-stained tissue, and positive staining in immunohistochemical-stained tissue. HE, hematoxylin/eosin; PAS, periodic acid-Schiff; MGA, mean glomerular area; MGV, mean glomerular volume, MGV = β/K x (MGA)^3/2^; NK/NC, normal control, *n* = 15; STZ, high-fat diet- and streptozotocin-treated, *n* = 15; CaD, calcium dobesilate, *n* = 15. *p < 0.05 *vs*. control group; ^#^ *p* < 0.05 *vs*. DKD (diabetic kidney disease) group.

The IPGTT was performed before the administration of CaD. The results indicated that the glucose tolerance is impaired in KK-Ay and HFD-induced diabetic mice (**Table 1**). To estimate the therapeutic effect of CaD, we measured the body weight (BW), FBG levels, kidney weight to body weight (KW/BW), urinary albumin excretion (UAE), serum creatinine (Scr) levels, urinary albumin/creatinine (ACR) levels, TG levels, TC levels, and alanine aminotransferase (ALT) levels of all groups after 12 weeks of treatment with CaD (*n* = 15) or vehicle (*n* = 15) (**Tables 2** and **3**, **Supplementary Materials**). **Figure 1A** shows the BW and blood glucose levels during 12 weeks of CaD or vehicle treatment. UAE levels and KW/BW in DKD were significantly decreased after CaD treatment. BW, FBG levels, KW/BW, ALT levels, TG levels, TC levels, and Scr levels were not significantly different before and after CaD treatment. Scr levels showed were not significantly different between DKD mice and normal control mice.

**Table 1:** Results of the intraperitoneal glucose tolerance test (IPGTT) in NC- and HFD-induced diabetic mice.

Glucose (mmol/L)	NC	HFD
0 min	6.73 ± 2.43	8.98 ± 2.92
15 min	21.30 ± 6.50	25.2 ± 7.30
30 min	18.17 ± 7.17	31.8 ± 4.20*
60 min	11.95 ± 4.95	27.83 ± 8.17*
90 min	11.10 ± 4.10	22.92 ± 5.72*
120 min	9.02 ± 3.12	19.97 ± 6.23*

NC, normal control, n = 15; HFD, high-fat diet, n = 15; * p < 0.05 vs. respective control.

**Table 2:** Results of the biochemical analysis of obese mice with spontaneous diabetes (KK-Ay) mice.

	NK	KK-Ay	KK-Ay+CaD
BW (g)	27.35 ± 1.37	44.23 ± 1.22*	43.22 ± 1.20*
FBG (mmol/L)	6.57 ± 1.81	30.30 ± 2.06*	30.90 ± 2.32*
KW/BW (mg/g)	4.47 ± 0.57	5.94 ± 0.26*	5.31 ± 0.33^#^
UAE (µg/24 h)	0.26 ± 0.10	11.23 ± 2.90*	7.11 ± 1.02^#^
ALT (U/L)	21.80 ± 7.80	60.66 ± 19.01	62.33 ± 17.09*
TG (mmol/L)	1.97 ± 0.35	3.42 ± 0.77*	3.16 ± 1.91*
TC (mmol/L)	1.20 ± 0.79	3.98 ± 2.1*	3.42 ± 1.56
Scr (mmol/L)	16.60 ± 9.09	16.58 ± 4.66	18.21 ± 4.22

BW, body weight; FBG, fasting blood glucose; KW/BW, kidney weight to body weight; UAE, urinary albumin excretion; ALT, alanine aminotransferase; TG, total triglycerides; TC, total cholesterol; Scr, serum creatinine; NK, normal control, n = 15; KK-Ay, n = 15; CaD, n = 15, calcium dobesilate. *p < 0.05 vs. NK group; ^#^p < 0.05 vs. KK-Ay group.

**Table 3:** Results of the biochemical analysis of HFD/streptozotocin (STZ)-induced diabetic mice.

	NC	HFD/STZ	HFD/STZ+CaD
BW (g)	23.49 ± 1.78	28.37 ± 2.07*	26.61 ± 1.37*
FBG (mmol/L)	6.37 ± 1.44	20.51 ± 3.44*	19.90 ± 5.33*
KW/BW (mg/g)	6.19 ± 0.51	7.79 ± 0.51*	6.41 ± 0.35^#^
UAE (µg/24 h)	0.01 ± 0.01	2.13 ± 0.30*	0.37 ± 0.31^#^
ALT (U/L)	23.81 ± 8.79	49.20 ± 2.10*	52.49 ± 3.01*
TG (mmol/L)	0.67 ± 0.31	3.13 ± 0.08*	3.04 ± 0.13*
TC (mmol/L)	1.49 ± 0.29	3.92 ± 1.15*	3.99 ± 0.80*
Scr (mmol/L)	11.51 ± 1.62	13.10 ± 3.01	12.79 ± 2.45

BW, body weight; FBG, fasting blood glucose; KW/BW, kidney weight to body weight; UAE, urinary albumin excretion; ALT, alanine aminotransferase; TG, total triglycerides; TC, total cholesterol; Scr, serum creatinine; NC, normal control, n = 15; HFD/STZ, high-fat diet- and streptozotocin-treated, n = 15; CaD, calcium dobesilate, n = 15. *p < 0.05 vs. NC group; ^#^p < 0.05 vs. HFD/STZ group.

### Calcium Dobesilate Attenuated Renal Histological Destruction

After 12 weeks of CaD treatment, the mice were sacrificed, and kidney samples were harvested. The size of the kidneys of KK-Ay (*n* = 15) and HFD/STZ (*n* = 15) mice was apparently larger than that of the kidneys of the NK (*n* = 15) and NC (*n* = 15) mice. The sizes were restored in the KK-Ay+CaD (*n* = 15) and HFD/STZ+CaD (*n* = 14) mice (**Figures 1B, C**). Meanwhile, HE, PAS, and Masson staining were performed to determine the MGA, MGV, MMA, and relative fibrotic area to measure the glomerular volume and evaluate kidney structure. The results showed that the MGA, MGV, MMA, and relative fibrotic area were increased in DKD and decreased after CaD treatment. These results indicate that CaD is capable of attenuating glomerular enlargement, glomerular MMA expansion, and glomerular and tubular fibrosis in DKD mice.

### Hyperglycemia-Induced Glomerular Filtration Membrane Injuries Were Restored by Calcium Dobesilate

To determine the effect of CaD on the glomerular filtration membrane, the glomerular-specific indicators nephrin and podocin were detected by immunohistochemistry (**Figures 1D, E**). In KK-Ay and HFD/STZ mice, the expression of nephrin and podocin was significantly lower than that in C57BL/6 mice, indicating more filtration membrane damage. CaD rescues the changes in DKD mice. The results confirmed the protective effect of CaD on the glomerulus. The results indicate that CaD is capable of reducing albuminuria and protecting against renal histological changes.

### Calcium Dobesilate Restored Autophagy in the Kidneys of KK-Ay and HFD/STZ Mice

**Figure 2:**
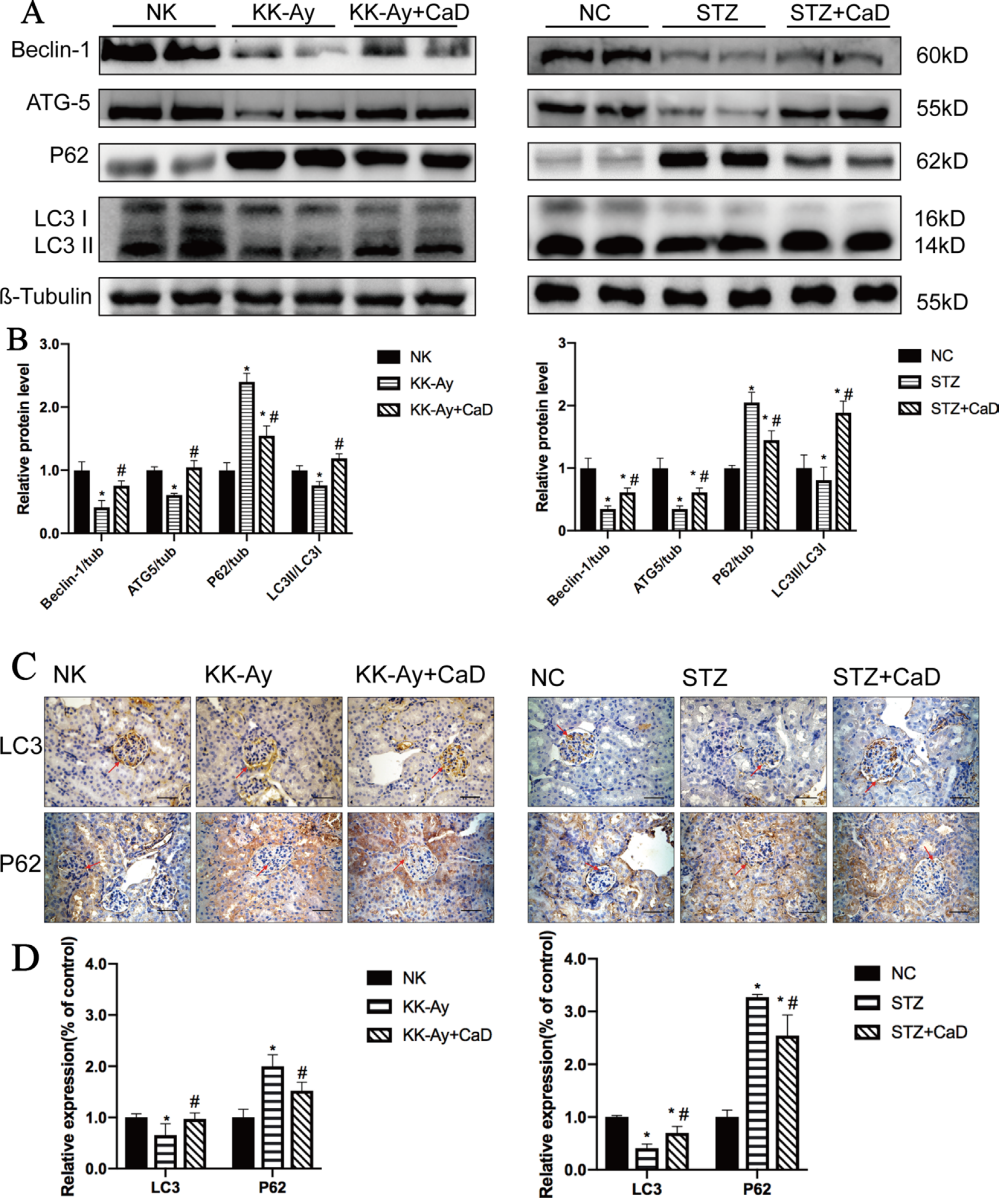
Calcium dobesilate upregulated autophagy in DKD mice. **(A)** Representative western blot analysis of Beclin 1, Atg5, p62, and LC3 in mice. **(B)** The densitometric results of Beclin 1, Atg5, P62, and LC3 expression, as determined by western blotting. The error bars represent the SEM. **(C, D)** The immunohistochemical quantification and analysis of LC3 and p62 in kidney samples. The scale bars in the right lower corner represent 100 µm. The arrows indicate positive staining. NK/NC: normal control, *n* = 15; STZ, high-fat diet- and streptozotocin-treated, *n* = 15; CaD: calcium dobesilate, *n* = 15. *p < 0.05 *vs*. control group; ^#^ *p* < 0.05 *vs*. DKD group.

We analyzed the abundance of autophagy-related proteins in kidney lysates by western blotting and immunohistochemistry. As shown in **Figures 2A, B**, the protein expression levels of LC3 II, Atg5, and beclin 1 were significantly decreased in the DKD groups compared with the respective control groups and significantly increased after CaD treatment. Moreover, the protein expression level of p62 was significantly increased in the DKD groups and decreased in the CaD treatment groups (**Figures 2A, B**). According to the immunohistochemistry results, the expression of LC3 was significantly decreased in the DKD groups compared with the control groups and restored in the CaD treatment groups. Meanwhile, the expression of p62 in DKD decreased after CaD treatment (**Figures 2C, D**). The results suggest that CaD is effective at restoring autophagy in DKD.

### Calcium Dobesilate Decreased VEGF and Downregulated VEGFR2

**Figure 3:**
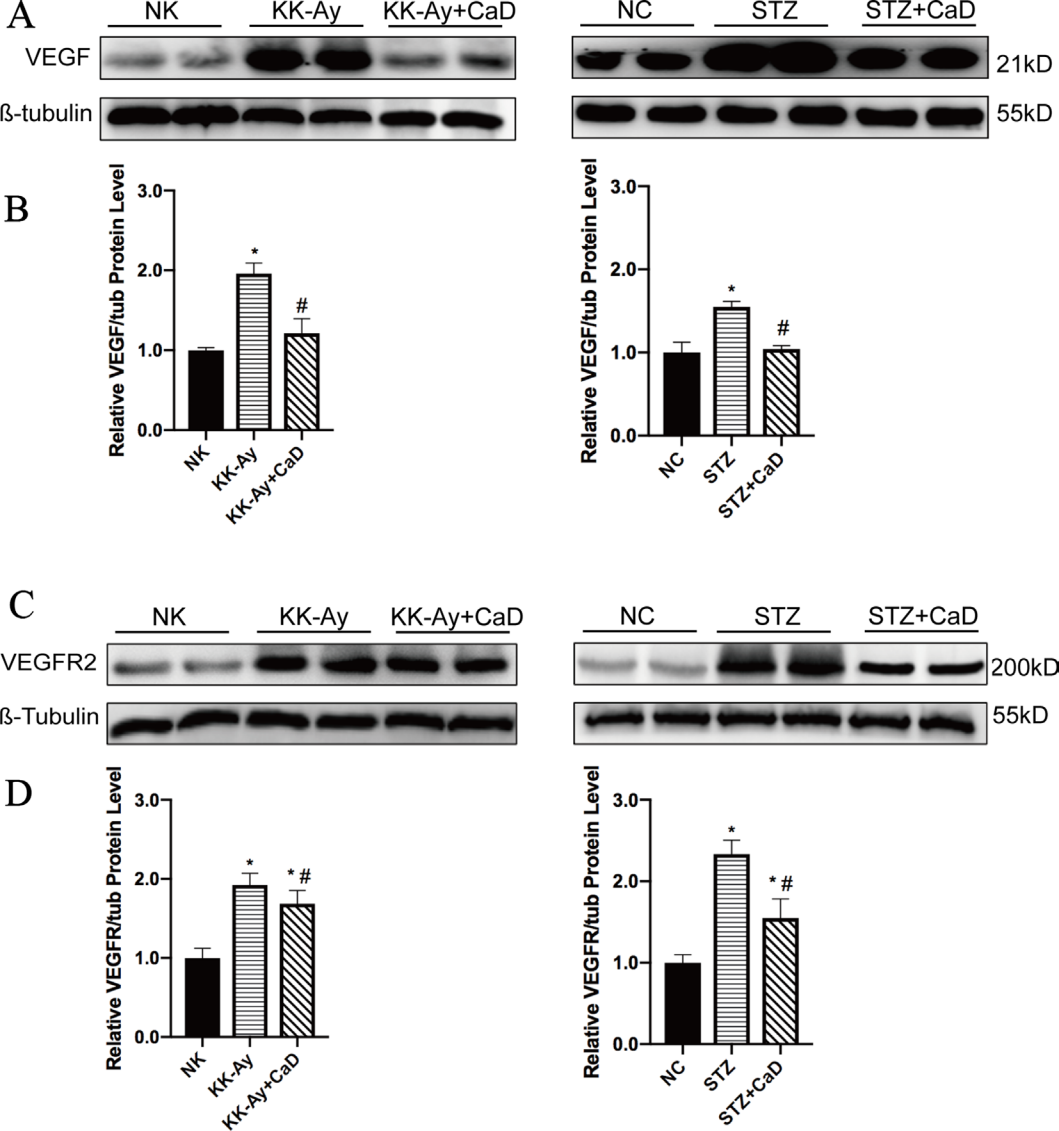
Calcium dobesilate decreased VEGF and VEGFR2. **(A)** Western blots of VEGF for each group. **(B)** Densitometric results of VEGF as determined by western blot. Error bars represent SEM. **(C)** Western blots of VEGFR2 for each group. **(D)** The densitometric results of VEGFR2 expression, as determined by western blot. The error bars represent the SEM. VEGF, vascular endothelial growth factor; VEGFR2, vascular endothelial growth factor receptor 2; NK/NC: normal control, *n* = 15; STZ, high-fat diet- and streptozotocin-treated, *n* = 15; CaD: calcium dobesilate, *n* = 15. *p < 0.05 *vs*. control group; ^#^ *p* < 0.05 *vs*. DKD group.

According to the western blotting analysis shown in **Figure 3**, the protein expression levels of VEGF and VEGFR2 were significantly increased in the KK-Ay and HFD/STZ groups compared with the control group. After CaD treatment, the expression levels of VEGF and VEGFR2 were restored. The results indicate that CaD is capable of restoring VEGF changes and maintaining vascular construction and function.

### Calcium Dobesilate Inactivated the PI3K/AKT/mTOR Signaling Pathway *via* Inhibiting the Phosphorylation of Proteins

**Figure 4:**
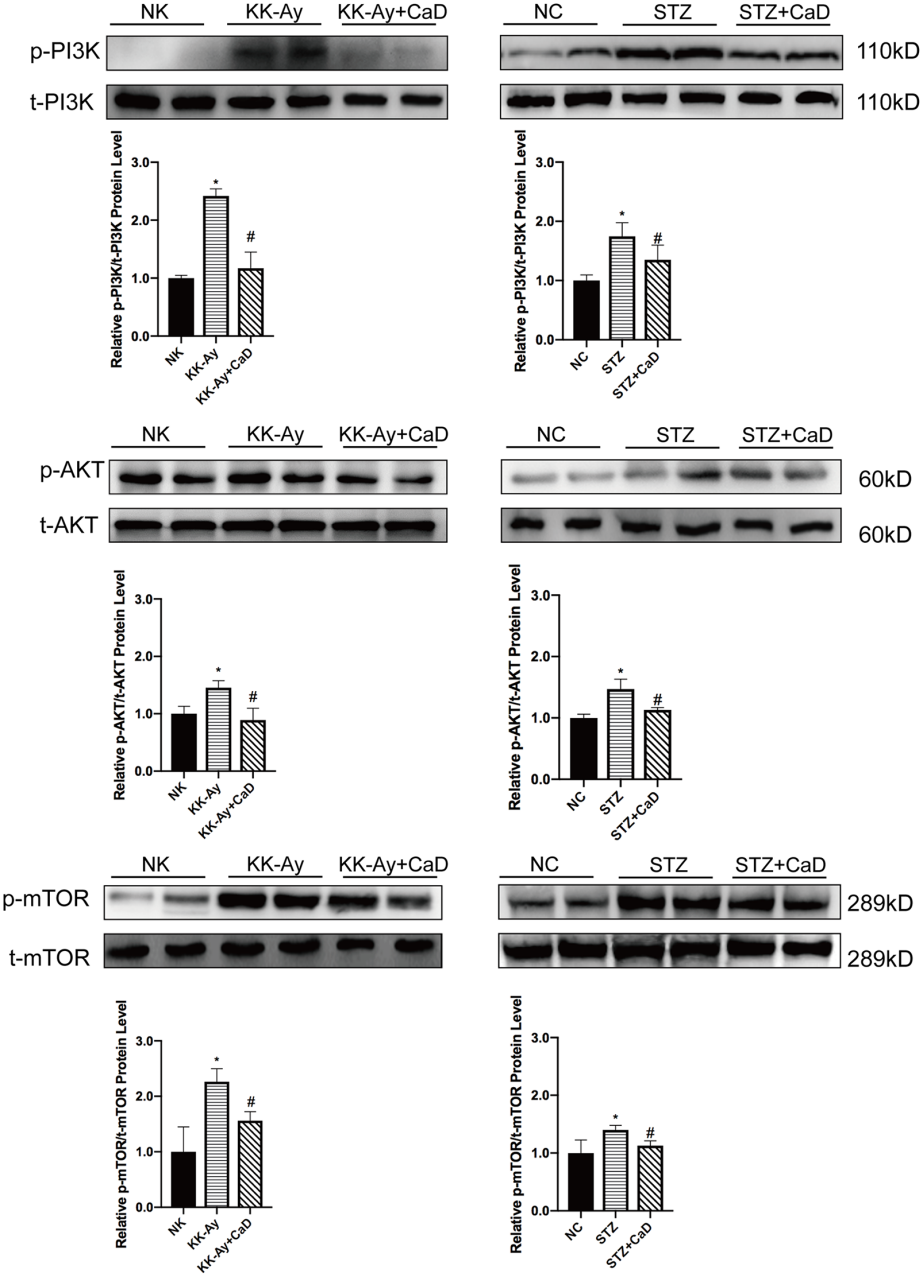
Calcium dobesilate inhibited the downstream of VEGF/VEGFR2/PI3K/AKT/mTOR signaling pathway. **(A)** Representative western blots of p-PI3K and t-PI3K. **(B)** The densitometric results of p-PI3K and t-PI3K expression, as determined by western blot. The error bars represent the SEM. **(C)** Representative western blots of p-Akt and t-Akt. **(D)** The densitometric results of p-Akt and t-Akt expression, as determined by western blot. The error bars represent the SEM. **(E)** Representative western blots of p-mTOR and t-mTOR. **(F)** The densitometric results of p-mTOR and t-mTOR expression, as determined by western blot. The error bars represent the SEM. p, phosphorylated; t, total; NK/NC: normal control, *n* = 15; STZ, high-fat diet- and streptozotocin-treated, *n* = 15; CaD: calcium dobesilate, *n* = 15. *p < 0.05 *vs*. control group; ^#^ *p* < 0.05 *vs*. DKD group.

**Figure 5:**
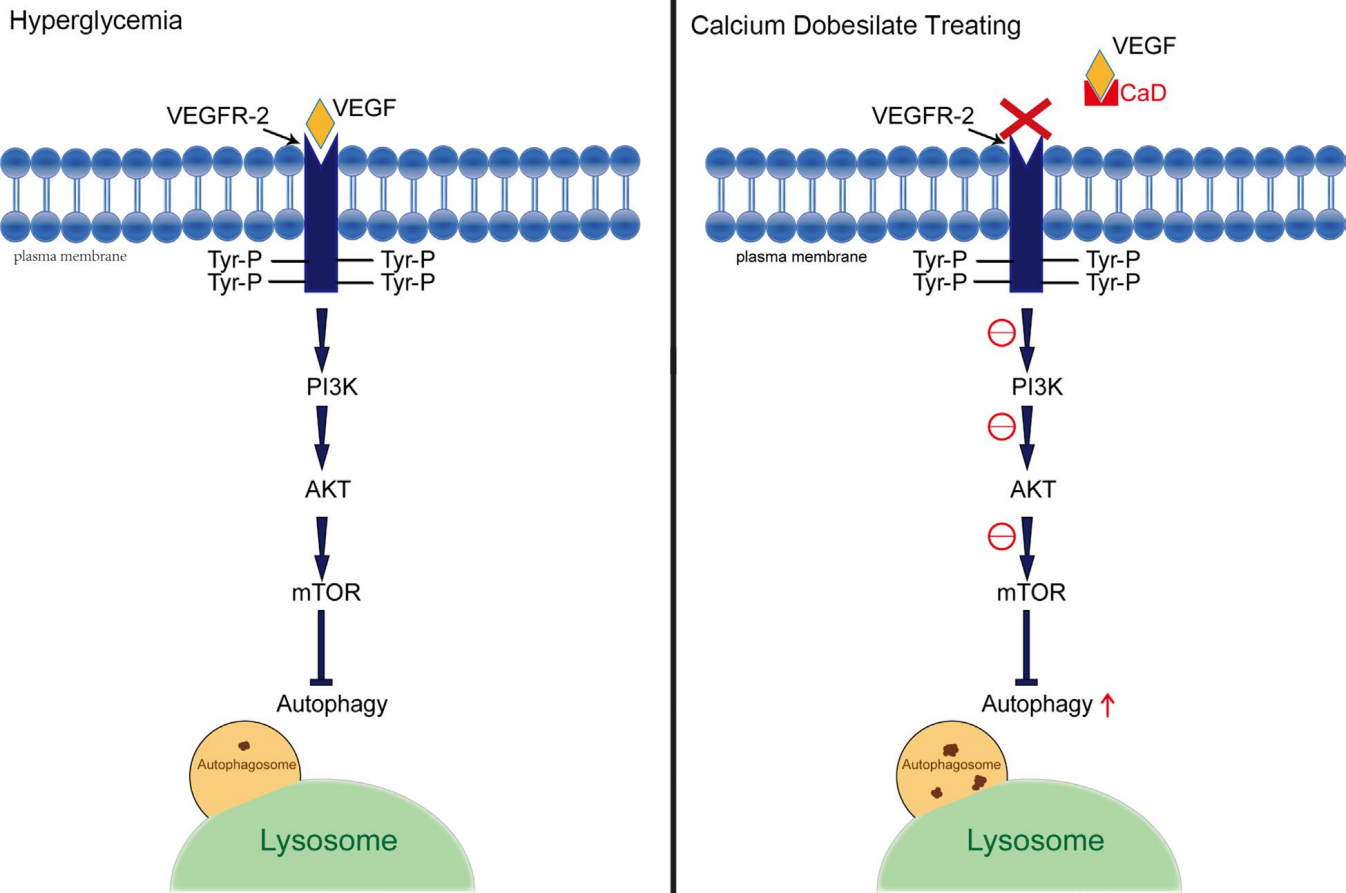
Hypothetical pathway between autophagy and VEGF. A presentation of the interplay between the implicated signaling factors under hyperglycemia and CaD therapy conditions. VEGF, vascular endothelial growth factor; VEGFR2, vascular endothelial growth factor receptor 2; Tyr-P, phosphorylated tyrosinase; CaD, calcium dobesilate.

To confirm the underlying mechanism of improved autophagy in CaD, we evaluated the activation of the PI3K/AKT/mTOR signaling pathway. As one of the downstream signaling pathways of VEGF/VEGFR2, PI3K/AKT, like VEGF/VEGFR2, is active under hyperglycemic conditions. According to the western blot results, the relative expression of p-PI3K/t-PI3K, p-AKT/t-AKT, and p-mTOR/t-AKT was increased in the DKD groups and decreased in the CaD treatment groups (KK-Ay+CaD and HFD/STZ+CaD) (**Figures 4A–F**). These results suggest that CaD inhibits the PI3K/AKT/mTOR pathway. Finally, we constructed a hypothetical pathway of CaD intervention (**Figure 5**).

## Discussion

In our study, we first demonstrated the ability of CaD to decrease albuminuria by restoring autophagy through the VEGF/PI3K/Akt/mTOR pathway in KK-Ay mice and HFD/STZ-induced diabetic mice. In diabetic murine models, CaD reduced KW/BW and UAE levels without altering BW, or ALT, Scr, TG, or TC levels. The results indicate that CaD may not be a hypoglycemic drug but may be useful for reducing albuminuria. It has been confirmed that CaD is protective for renal function and is has a potential clinical application for delaying DKD progression.

Two diabetic models were used in our study: obese mice with spontaneous diabetes (KK-Ay) and HFD/STZ. An ideal model of diabetic nephropathy should exhibit progressive albuminuria, abnormal renal function, and characteristic histological changes in the glomeruli and tubulointerstitial lesions (Breyer et al., 2005; Dusane and Joshi, 2013; Xiao et al., 2018). KK-Ay mice are a typical animal model of the early to moderate stages of type 2 diabetic nephropathy (Kitada et al., 2016).

Additionally, to exclude the specificity of animal models and make the research more convincing, we also used HFD/STZ-induced diabetic mice. HFD feeding is known to induce various systemic metabolic alterations, including obesity, insulin resistance, hyperglycemia, and abnormal lipid metabolism. Systematic lipid overload and glycemia may explain renal injury (Malatiali et al., 2008; Furman, 2015). STZ is known for its toxicity, especially on islet β cells. According to our results, renal injury, including glomerular hypertrophy, mesangial matrix expansion, and the thickening of the glomerular and tubular basement membrane, are more severe in HFD/STZ mice than in KK-Ay mice. Hyperglycemia-induced oxidative stress and STZ toxicity may account for the results.

On the basis of the results of albuminuria levels, the GMV, the MMA, and IH for nephrin and podocin, CaD significantly restored glomerular, mesangial, and tubular lesions. Thus, we hypothesized that CaD is angioprotective and is a promising candidate for the treatment of chronic kidney diseases.

Albuminuria is usually considered to be representative of an injured filtration membrane and decreased renal function (Retnakaran et al., 2006). The pathophysiology includes altered hemodynamics, ischemia, inflammation, overactive RAAS, and oxidative stress. Recent studies have shown that the novel pathogenesis of DKD includes genetic modification, epigenetic regulation, decreased podocyte autophagy, and mitochondrial dysfunction (Lin et al., 2018). In conclusion, renal injury is caused by various factors. Thus, we aimed to determine the alterations in autophagy that occur during DKD progression and to determine whether CaD alters autophagy. The quantitative level of autophagy can be determined by the gray values of LC3 II/LC3 I, p62/β-tubulin, beclin 1/β-tubulin, and ATG5/β-tubulin bands (Gordy and He, 2012; Parzych and Klionsky, 2014; Tsapras and Nezis, 2017) (**Figure 2**). Our results confirm this conjecture. CaD significantly restored autophagy in DKD.

CaD is applied for the treatment of diabetic microvascular diseases, especially for diabetic retinopathy because of its angioprotective properties (Suschek et al., 1997; Brunet et al., 1998; Brunet et al., 1998; Leal et al., 2010). Clinical studies have noted that alprostadil combined with CaD is widely applied for the treatment of renal disease (Qin et al., 2017), and CaD has been confirmed to be effective in maintaining the construction of the vascular endothelium and enhancing renal function (John and Ning, 2016). However, the specific mechanism is not yet clear. One recent study demonstrated that ferulic acid can circumvent oxidative stress-mediated renal cell damage by regulating autophagy (Lei et al., 2018; Chowdhury et al., 2019). In our study, we demonstrated for the first time that CaD is effective in restoring autophagy in the kidneys of KK-Ay and HFD/STZ-induced diabetic mice. The restored autophagy may account for the decreased albuminuria level in DKD mice after CaD treatment through repairing the filtration membranes.

In addition, VEGF disturbance is also a major factor that results in renal injury by inducing angiogenesis and vascular permeability. VEGF is overexpressed under stress conditions such as hyperglycemia. Excessive VEGF participates in the pathogenesis of endothelial cell dysfunction. VEGF acts by phosphorylating tyrosine residues of the VEGF receptor and regulates incomputable downstream effectors by cooperating with compensatory signaling pathways (Hiratsuka et al., 2001). VEGFR1 is primarily involved in macrophage immigration and tumor angiogenesis. VEGFR2 is mainly expressed in the endothelium and participates in the regulation of endothelial cell performance. VEGFR3 is mainly involved in lymphangiogenesis (Feng et al., 1999; Claesson-Welsh, 2016).

Most of the cellular actions of VEGF in endothelial cells (e.g., the regulation of vascular permeability and the fenestration of endothelial cells) are predominantly regulated by VEGFR2 (Roberts and Palade, 1997; Lohela et al., 2009), and some studies have demonstrated that VEGF promotes eNOS phosphorylation *via* VEGFR2 in GENCs, resulting in an enhanced generation of nitric oxide (Feliers et al., 2005). In summary, overexpressed VEGF is harmful for maintaining the structure of glomerular filtration barriers. In our present study, CaD significantly reduced the expression of VEGF and downregulated VEGFR2. Simultaneously, CaD restored filtration membrane injury markers (nephrin and podocin) and albuminuria levels. Therefore, we assume that the alleviation of renal injuries by CaD may have been due to reduced VEGF action. In other words, the angioprotective effect of CaD was confirmed.

The VEGF family mainly includes VEGF-A, VEGF-B, VEGF-C, VEGF-D, VEGF-E, and placenta growth factor (PIGF). VEGF-A binds both VEGFR1 and VEGFR2, whereas VEGF-B and PlGF bind only VEGFR1 (Maglione et al., 1991; de Vries et al., 1992; Terman et al., 1992). Activated VEGF-C/D bind and activate VEGFR2 in addition to VEGFR3 (Joukov et al., 1996). Among the members of the VEGF family, VEGF-A plays a principal role in regulating vascular permeability and producing eNOS (Shibuya and Claesson-Welsh, 2006; Haller et al., 2017). We did not determine which ligands are involved in the alleviation of autophagy and renal injury by CaD in this study. Previous studies have shown that CaD acts as a heparin antagonist within the binding site and changes the three-dimensional structure of the growth factor at its receptor-recognizing site. As a result, the receptor-growth factor signaling complex is dissociated (Haller et al., 2017). In other words, CaD inhibits the binding of VEGF to heparin sulfates, thereby reducing the affinity of the VEGF/heparin sulfate complex for its receptor-binding sites.

While the effect of CaD on downregulating the activation of VEGF/VEGFR2 and restoring autophagy was confirmed, the specific mechanism is still unclear. In our study, we demonstrated that one of the possible pathways, the PI3K/Akt/mTOR pathway, was affected by CaD. Three main downstream pathways of VEGF/VEGFR2 exist: the JAK/STAT, MEK/ERK, and PI3K/AKT pathways. It has been demonstrated that phosphoinositide 3-kinase (PI3K) is induced by VEGF/VEGFR2, which augments PI3K activation by phosphorylation. VEGF also activates the AKT pathway in a PI3K-dependent manner (Ruan and Kazlauskas, 2012; Feliers et al., 2005). In our study, phosphorylated PI3K and AKT were significantly decreased after CaD treatment in KK-Ay and HFD/STZ-induced diabetic mice. As one of the downstream effectors of PI3K/AKT, mTOR is closely related to autophagy (Zoncu et al., 2011; Dunlop and Tee, 2014).

The primary signaling pathways that regulate autophagy include the mTOR, AMP-activated protein kinase (AMPK), and sirtuin (SIRT) pathways. We examined changes in AMPK phosphorylation before and after CaD treatment in DKD. There were no significant differences. Thus, we concluded that CaD rarely acts on the AMPK signaling pathway. mTOR phosphorylation decreased after CaD treatment in KK-Ay and HFD/STZ-induced diabetic mice. Thus, we concluded that CaD may regulate autophagy in DKD by inhibiting the PI3K/AKT/mTOR pathway, which is a downstream pathway of VEGFR2. However, there may be more links between VEGF and autophagy, and further research is required.

In conclusion, the angioprotective and autophagy-restoring effects of CaD were demonstrated in our study. Currently, CaD is mainly used for the treatment of diabetic retinopathy because it is effective in maintaining blood-retinal barrier integrity and reducing the permeability of the retina (Brunet et al., 1998), which is possibly associated with autophagy. According to those properties, CaD is a very promising candidate for treating other types of autophagy-related organ damage and complications of vascular injury.

## Data Availability

The raw data supporting the conclusions of this manuscript will be made available by the authors, without undue reservation, to any qualified researcher.

## Ethics Statement

This study was carried out in accordance with the Animal Use Guidelines of the university committee. The protocol was approved by the Animal Use Committee of Tianjin Medical University.

## Author Contributions

C-jL, BS and L-mC conceived the project. YW and Y-hL performed the experiments and wrote the entire manuscript. CT, X-cY, Y-pC, MX, X-yL, TL and YC participated in part of the work. All authors read and approved the final manuscript.

## Funding

This work was supported by the National Natural Science Foundation of China (No. 81470187), the Natural Science Foundation of Tianjin (Nos. 15ZXHLSY00460, 18JCYBJC26100 and 18JCZDJC35500), and the Bethune-Merck Fund to C-JL.

## Conflict of Interest Statement

The authors declare that the research was conducted in the absence of any commercial or financial relationships that could be construed as a potential conflict of interest.

## References

[B1] Advani A. Kelly D. J. Advani S. L. Cox A. J. Thai K. Zhang Y. (2007). Role of VEGF in maintaining renal structure and function under normotensive and hypertensive conditions. Proc. Natl. Acad. Sci. 104 (36), 14448–14453. 10.1073/pnas.0703577104 17726104 PMC1964850

[B2] Angulo J. Peiró C. Romacho T. Fernández A. Cuevas B. González-Corrochano R. (2011). Inhibition of vascular endothelial growth factor (VEGF)-induced endothelial proliferation, arterial relaxation, vascular permeability and angiogenesis by dobesilate. Eur. J. Pharmacol. 667 (1-3), 153–159. 10.1016/j.ejphar.2011.06.015 21703259

[B3] Breyer M. D. Böttinger E. Brosius F. C. Coffman T. M. Sharma K. (2005). Mouse models of diabetic nephropathy. J. Am. Soc. Nephrol. 16 (1), 27–45. 10.1681/ASN.2004080648 15563560

[B4] Brunet J. Farine J. C. Garay R. P. Hannaert P. (1998). Angioprotective action of calcium dobesilate against reactive oxygen species-induced capillary permeability in the rat. Eur. J. Pharmacol. 358 (3), 213–220. 10.1016/S0014-2999(98)00604-9 9822887

[B5] Brunet J. Farine J. C. Garay R. P. Hannaert P. (1998). *In vitro* antioxidant properties of calcium dobesilate. Fundam. Clin. Pharmacol. 12 (2), 205–212. 10.1111/j.1472-8206.1998.tb00943.x 9565776

[B6] Chowdhury S. Ghosh S. Das A. K. Sil P. C. (2019). Ferulic acid protects hyperglycemia-induced kidney damage by regulating oxidative insult, inflammation and autophagy. Front. Pharmacol. 10, 27. 10.3389/fphar.2019.00027 30804780 PMC6371841

[B7] Claesson-Welsh L. (2016). Vegf receptor signal transduction– a brief update. Vasc. Pharmacol. 86, 14–17. 10.1016/j.vph.2016.05.011 27268035

[B8] Collins A. J. Foley R. N. Chavers B. (2012). United states renal data system 2011 annual data report: atlas of chronic kidney disease & end-stage renal disease in the United States. Am. J. Kidney Dis. 59, A7e1–A420. 10.1053/j.ajkd.2011.11.015 22177944

[B9] Cooper M. E. Vranes D. Youssef S. Stacker S. A. Cox A. J. Rizkalla B. (1999). Increased renal expression of vascular endothelial growth factor (VEGF) and its receptor VEGFRR-2 in experimental diabetes. Diabetes 48 (11), 2229–2239. 10.2337/diabetes.48.11.2229 10535459

[B10] de Vries C. Escobedo J. A. Ueno H. Houck K. Ferrara N. Williams L. T. (1992). The fms-like tyrosine kinase, a receptor for vascular endothelial growth factor. Science 255 (5047), 989–991. 10.1126/science.1312256 1312256

[B11] Dunlop E. A. Tee A. R. (2014). mTOR and autophagy: a dynamic relationship governed by nutrients and energy. Semin. Cell Dev. Biol. 36, 121–129. 10.1016/j.semcdb.2014.08.006 25158238

[B12] Dusane M. B. Joshi B. N. (2013). Beneficial effect of flax seeds in streptozotocin (STZ) induced diabetic mice: isolation of active fraction having islet regenerative and glucosidase inhibitory properties. Can. J. Physiol. Pharmacol. 91, 325–331. 10.1139/cjpp-2011-0428 23656171

[B13] Feliers D. Chen X. Aki N. Choudhury G. G. Madaio M. Kasinath B. S. (2005). Vegf regulation of endothelial nitric oxide synthase in glomerular endothelial cells. Kidney Int. 68 (4), 1648–1659. 10.1111/j.1523-1755.2005.00575.x 16164642

[B14] Feng D. Nagy J. A. Pyne K. Hammel I. Dvorak H. F. Dvorak A. M. (1999). Pathways of macromolecular extravasation across microvascular endothelium in response to vpf/vegf and other vasoactive mediators. Microcirculation 6 (1), 23–44. 10.1080/713773925 10100187

[B15] Ferrara N. Gerber H. P. Lecouter J. (2003). The biology of VEGF and its receptors. Nat. Med. 9 (6), 669–676. 10.1038/nm0603-669 12778165

[B16] Furman B. L. (2015). Streptozotocin-induced diabetic models in mice and rats. Curr. Protoc. Pharmacol. 70, 5471. 10.1002/0471141755.ph0547s70 26331889

[B17] Gordy C. He Y. W. (2012). The crosstalk between autophagy and apoptosis: where does this lead? Protein & Cell 3 (1), 17–27. 10.1007/s13238-011-1127-x 22314807 PMC4875212

[B18] Haller H. Ji L. Stahl K. Bertram A. Menne J. (2017). Molecular mechanisms and treatment strategies in diabetic nephropathy: new Avenues for calcium dobesilate- free radical scavenger and growth factor inhibition. Biomed. Res. Int. 2017, 1–11. 10.1155/2017/1909258 PMC5634607 29082239

[B19] Hiratsuka S. Maru Y. Okada A. Seiki M. Shibuya M. (2001). Involvement of flt-1 tyrosine kinase (vascular endothelial growth factor receptor-1) in pathological angiogenesis. Cancer Res. 61 (3), 1207–1213. 10.1097/00002820-200102000-00011 11221852

[B20] John S. Ning L. X. (2016). Complication in diabetic nephropathy. Diabetes Metab. Syndr. Clin. Res. Rev. 10 (4), 247–249. 10.1016/j.dsx.2016.06.005 27389078

[B21] Joukov V. Pajusola K. Kaipainen A. Chilov D. Lahtinen I. Kukk E. (1996). A novel vascular endothelial growth factor, VEGF-C, is a ligand for the Flt4 (VEGFR- 3) and KDR (VEGFR-2) receptor tyrosine kinases. EMBO J. 15 (7), 1751. 10.1002/j.1460-2075.1996.tb00521.x 8612600 PMC450088

[B22] Kitada M. Yoshio O. Daisuke K. (2016). Rodent models of diabetic nephropathy: their utility and limitations. Int. J. Nephrol. Renovasc. Dis. 9, 279–290. 10.2147/IJNRD.S103784 27881924 PMC5115690

[B23] Leal E. C. Martins J. Voabil P. Liberal J. Chiavaroli C. Bauer J. (2010). Calcium dobesilate inhibits the alterations in tight junction proteins and leukocyte adhesion to retinal endothelial cells induced by diabetes. Diabetes 59 (10), 2637–2645. 10.2337/db09-1421 20627932 PMC3279541

[B24] Lei L. Ying J. Ravindran J. Yanli H. (2018). Current advances in pharmacotherapy and technology for diabetic retinopathy: a systematic review. J. Ophthalmol. 2018, 1–13. 10.1155/2018/1694187 PMC5822768 29576875

[B25] Lenoir O. Jasiek M. Hénique C. Guyonnet L. Hartleben B. Bork T. (2015). Endothelial cell and podocyte autophagy synergistically protect from diabetes-induced glomerulosclerosis. Autophagy 11 (7), 1130–1145. 10.1080/15548627.2015.1049799 26039325 PMC4590611

[B26] Lin Y. C. Chang Y. H. Yang S. Y. Wu K. D. Chu T. S. (2018). Update of pathophysiology and management of diabetic kidney disease. J. Formos. Med. Assoc. 177 (8), 662–675. 10.1016/j.jfma.2018.02.007 29486908

[B27] Lohela M. Bry M. Tammela T. Alitalo K. (2009). VEGFs and receptors involved in angiogenesis versus lymphangiogenesis. Curr. Opin. Cell Biol. 21 (2), 154–165. 10.1016/j.ceb.2008.12.012 19230644

[B28] Maglione D. Guerriero V. Viglietto G. Persico D. B. G. (1991). Isolation of a human placenta cDNA coding for a protein related to the vascular permeability factor. Proc. Natl. Acad. Sci. U.S.A. 88 (20), 9267–9271. 10.1073/pnas.88.20.9267 1924389 PMC52695

[B29] Malatiali S. Francis I. Barac-Nieto M. (2008). Phlorizin prevents glomerular hyperfiltration but not hypertrophy in diabetic rats. Exp. Diabetes Res. 2008, 1–7. 10.1155/2008/305403 PMC2522335 18769499

[B30] Malatiali S. Francis I. Baracnieto M. (2016). Insulin prevents hyperfiltration and proteinuria but not glomerular hypertrophy and increases mesangial matrix expansion in diabetic rats. Med. Princ. Pract. 26 (1), 78–83. 10.1159/000450864 27643698 PMC5588318

[B31] McMahon A. M. Carati C. J. Piller N. B. Gannon B. J. (1994). The effects of radiation on the contractile activity of guinea pig mesenteric lymphatics. Lymphology 27, 193–200. 10.1580/0953-9859-5.4.455 7898134

[B32] Parzych K. R. Klionsky D. J. (2014). An overview of autophagy: morphology, mechanism, and regulation. Antioxid. Redox Signal. 20 (3), 460–473. 10.1089/ars.2013.5371 23725295 PMC3894687

[B33] Qin L. Qin W. Wang J. Lin L. (2017). Combined treatment of diabetic nephropathy with alprostadil and calcium dobesilate. Exp. Ther. Med. 14 (5), 5012–5016. 10.3892/etm.2017.5115 29201206 PMC5704275

[B34] Retnakaran R. Cull C. A. Thorne K. I. Adler A. I. Holman R. R. (2006). Risk factors for renal dysfunction in type 2 diabetes: U.K. prospective diabetes study 74. Diabetes 55 (6), 1832–1839. 10.2337/db05-1620 16731850

[B35] Risa W. Jun K. Daiki S. Azusa S. Kozo M. Akihiko M. (2017). 10-hydroxy-2-decenoic acid, a natural product, improves hyperglycemia and insulin resistance in obese/diabetic kk-ay mice, but does not prevent obesity. J. Vet. Med. Sci. 79 (9), 1596–1602. 10.1292/jvms.17-0348 28740028 PMC5627335

[B36] Roberts W. G. Palade G. E. (1997). Neovasculature induced by vascular endothelial growth factor is fenestrated. Cancer Res. 57 (4), 765–772. 9044858

[B37] Ruan G. X. Kazlauskas A. (2012). Vegf-a engages at least three tyrosine kinases to activate pi3k/akt. Cell Cycle 11 (11), 2047–2048. 10.4161/cc.20535 22647379 PMC3368856

[B38] Semenkovich K. Brown M. E. Svrakic D. M. Lustman P. J. (2015). Depression in type 2 diabetes mellitus: prevalence, impact, and treatment. Drugs 75 (6), 577–587. 10.1007/s40265-015-0347-4 25851098

[B39] Shibuya M. Claesson-Welsh L. (2006). Signal transduction by VEGF receptors in regulation of angiogenesis and lymphangiogenesis. Exp. Cell Res. 312 (5), 549–560. 10.1016/j.yexcr.2005.11.012 16336962

[B40] Suschek C. Kolb H. Kolb-Bachofen V. (1997). Dobesilate enhances endothelial nitric oxide synthase-activity in macro- and microvascular endothelial cells. Br. J. Pharmacol. 122 (7), 1502–1508. 10.1038/sj.bjp.0701512 9421302 PMC1565074

[B41] Terman B. I. Dougher-Vermazen M. Carrion M. E. Dimitrov D. Böhlen P. (1992). : Identification of the KDR tyrosine kinase as a receptor for vascular endothelial cell growth factor. Biochem. Biophys. Res. Commun. 187 (3), 1579–1586. 10.1016/0006-291X(92)90483-2 1417831

[B42] Tsapras P. Nezis I. P. (2017). Caspase involvement in autophagy. Cell Death Differ. 24 (8), 1369–1379. 10.1038/cdd.2017.43 28574508 PMC5520455

[B43] Villegas G. Lange-Sperandio B. Tufro A. (2005). Autocrine and paracrine functions of vascular endothelial growth factor (VEGF) in renal tubular epithelial cells. Kidney Int. 67 (2), 449–457. 10.1111/j.1523-1755.2005.67101.x 15673292

[B44] Xiao Y. Wu Q. Q. Duan M. X. Liu C. Yuan Y. Yang Z. (2018). Tax1bp1 overexpression attenuates cardiac dysfunction and remodeling in STZ-induced diabetic cardiomyopathy in mice by regulating autophagy. BBA-MOL BASIS DIS 18645 Pt A, 1728–1743. 10.1016/j.bbadis.2018.02.012 29476905

[B45] Yang W. Yu X. Zhang Q. Lu Q. Wang J. Cui W. (2013). Attenuation of streptozotocin-induced diabetic retinopathy with low molecular weight fucoidan *via* inhibition of vascular endothelial growth factor. Exp. Eye Res. 115 (Complete), 96–105. 10.1016/j.exer.2013.06.011 23810809

[B46] Yasuda-Yamahara M. Kume S. Tagawa A. Maegawa H. Uzu T. (2015). Emerging role of podocyte autophagy in the progression of diabetic nephropathy. Autophagy 11 (12), 2385–2386. 10.1080/15548627.2015.1115173 26565953 PMC4835189

[B47] Yin H. Wang W. Yu W. Li J. Feng N. Wang L. (2017). Changes in synaptic plasticity and glutamate receptors in type 2 diabetic kk-ay mice. J. Alzheimers Dis. 57 (4), 1207–1220. 10.3233/JAD-160858 28304288

[B48] Yoon M. S. Jankowski V. Montag S. Zidek W. Henning L. Schlüter H. (2004). Characterisation of advanced glycation endproducts in saliva from patients with diabetes mellitus. Biochem. Biophys. Res. Commun. 323 (2), 377–381. 10.1016/j.bbrc.2004.08.118 15369762

[B49] Zoncu R. Sabatini D. M. Efeyan A. (2011). mTOR: from growth signal integration to cancer, diabetes and ageing. Nat. Med. Mol. Cell 10.1038/nrm3025 PMC3390257 21157483

