# Supplementary figures and images for: Calcium Dobesilate Restores Autophagy by Inhibiting the VEGF/PI3K/AKT/mTOR Signaling Pathway
Source: Front Pharmacol. 2019 Aug 9;10:886. doi: 10.3389/fphar.2019.00886 (PMC6696883; doi:10.3389/fphar.2019.00886)

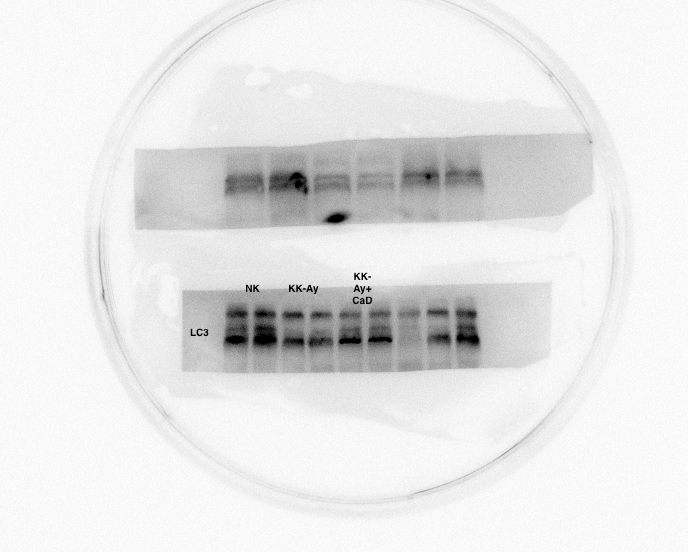

Supplement: Supplementary file 1 [file DataSheet_1.zip › original images of western blots/1.tiff]

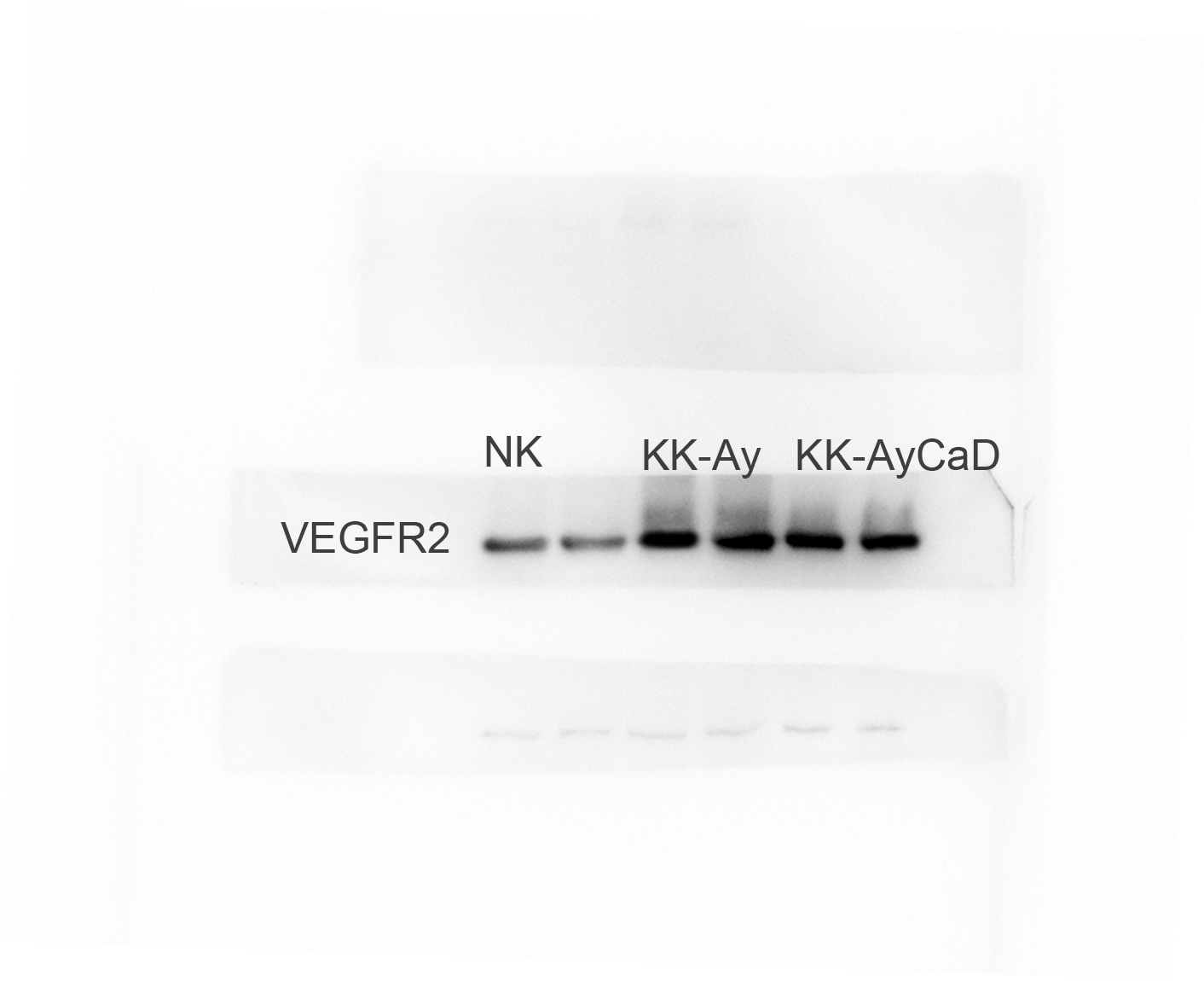

Supplement: Supplementary file 1 [file DataSheet_1.zip › original images of western blots/11.tiff]

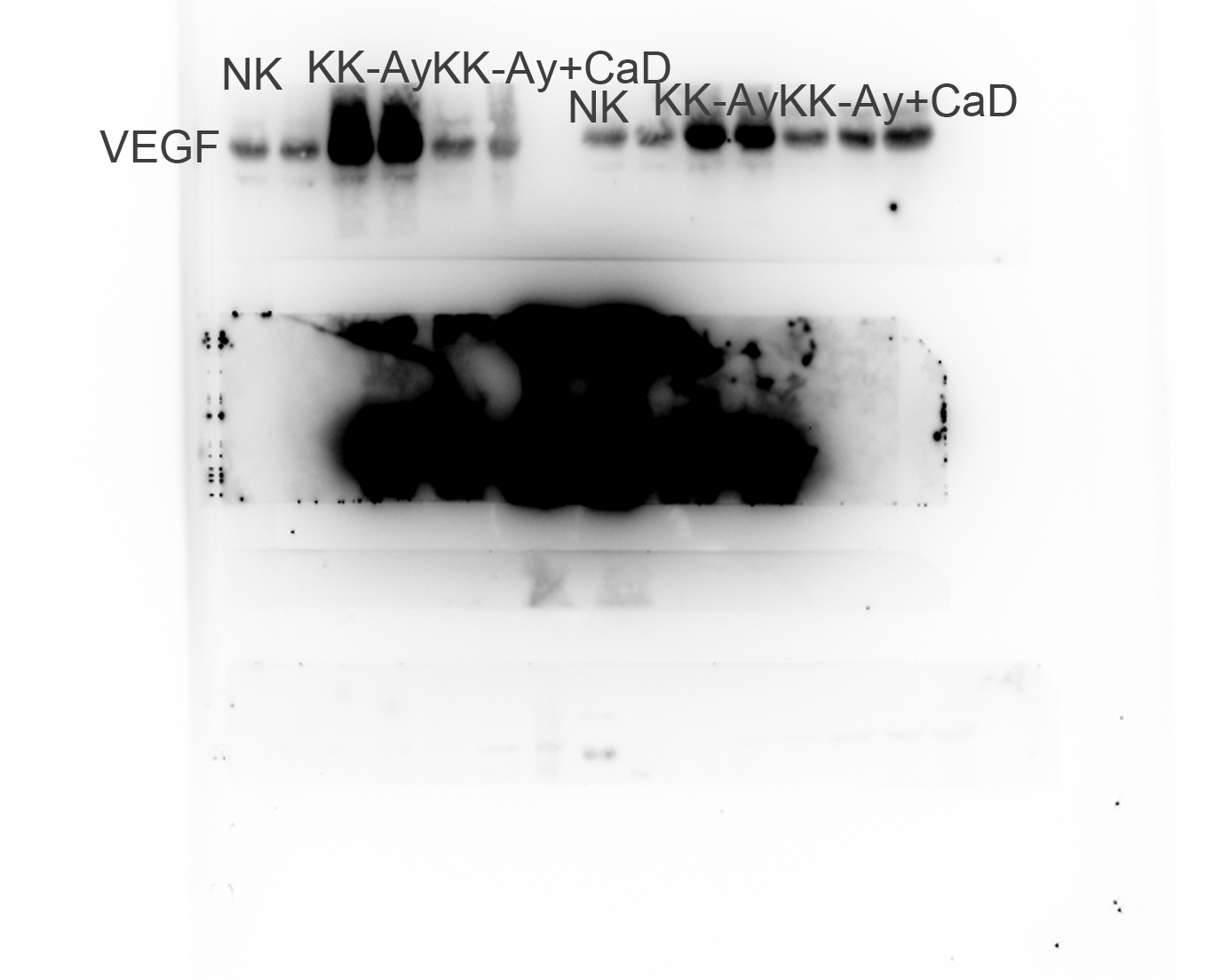

Supplement: Supplementary file 1 [file DataSheet_1.zip › original images of western blots/10.tiff]

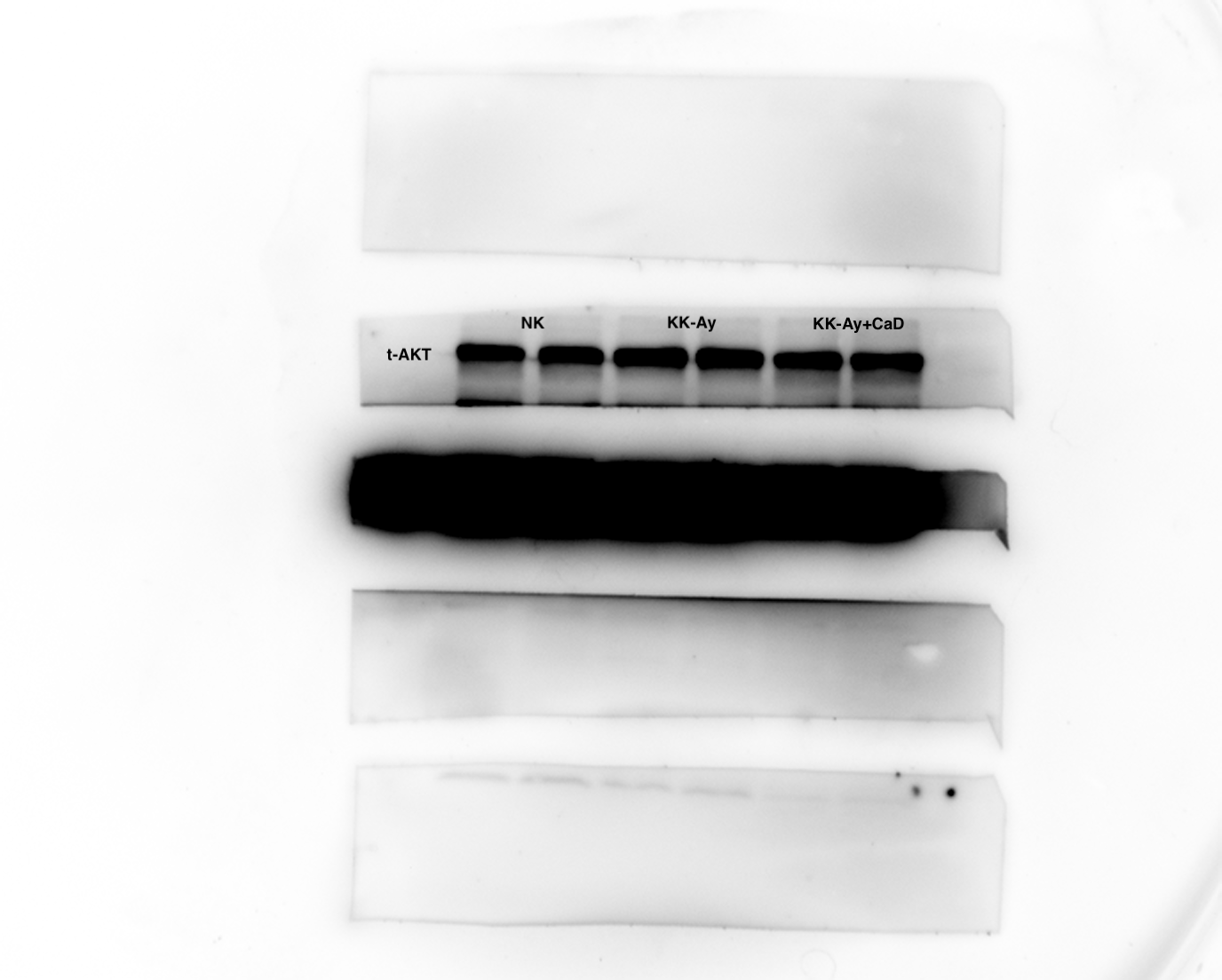

Supplement: Supplementary file 1 [file DataSheet_1.zip › original images of western blots/7.tiff]

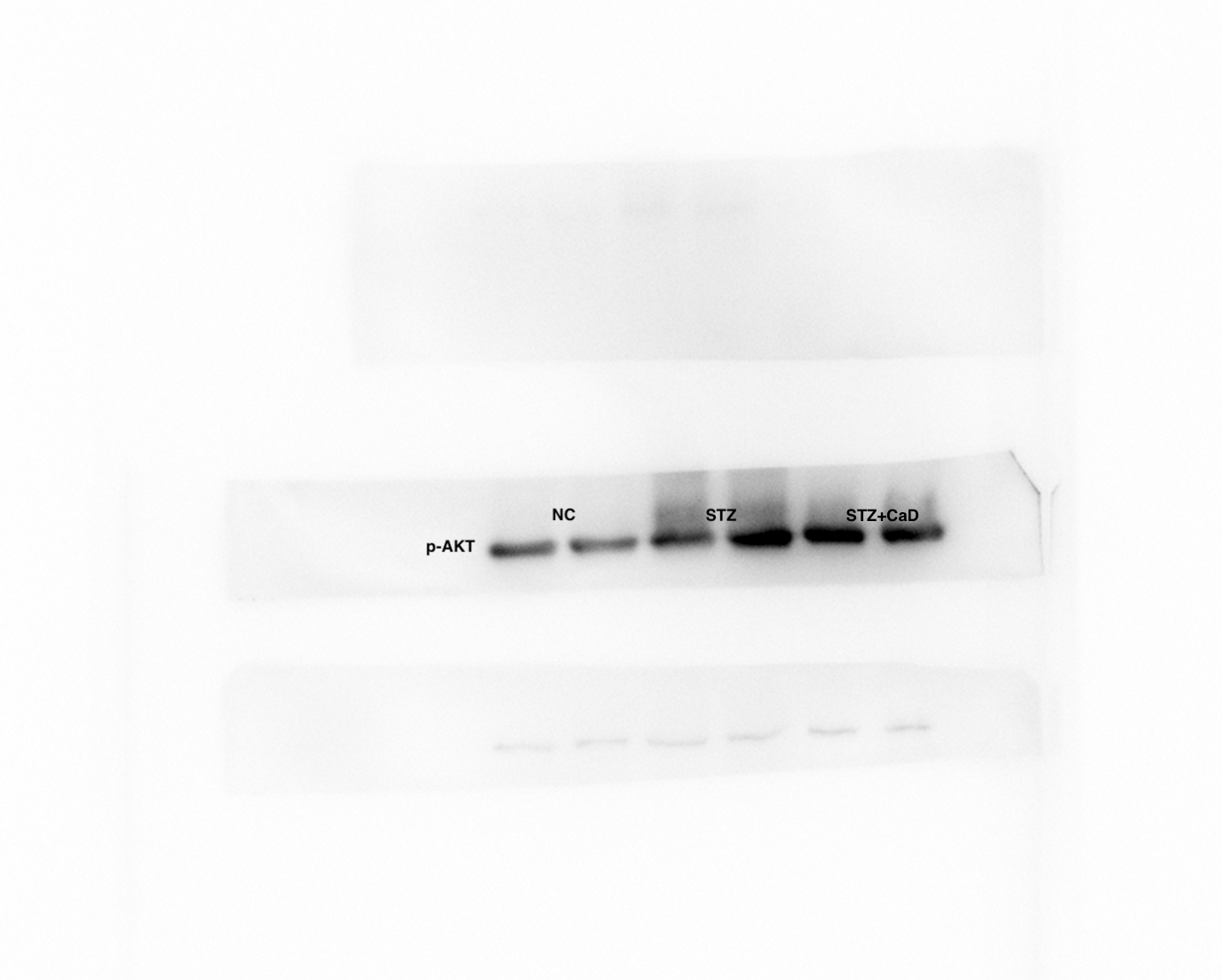

Supplement: Supplementary file 1 [file DataSheet_1.zip › original images of western blots/17.tiff]

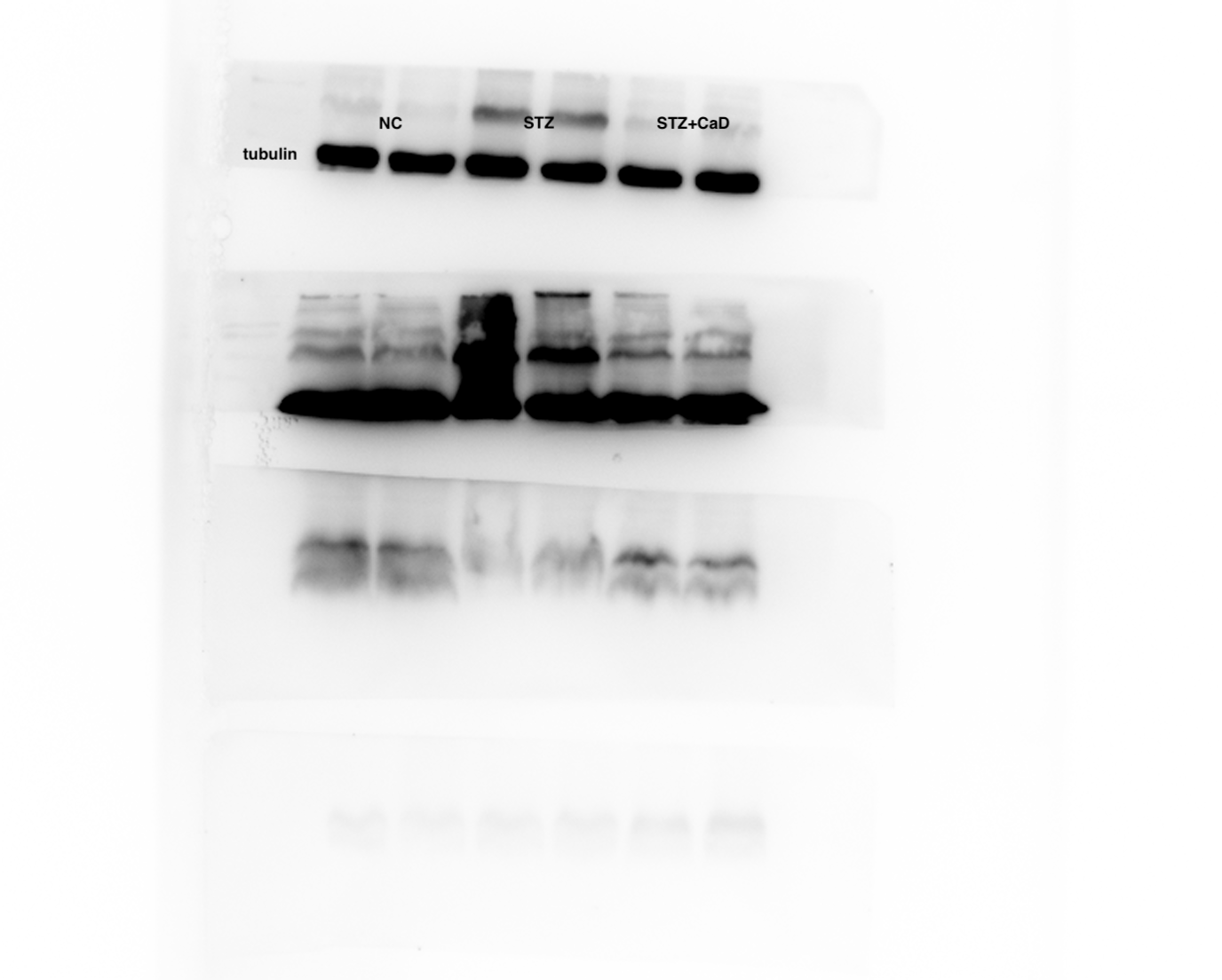

Supplement: Supplementary file 1 [file DataSheet_1.zip › original images of western blots/21.tiff]

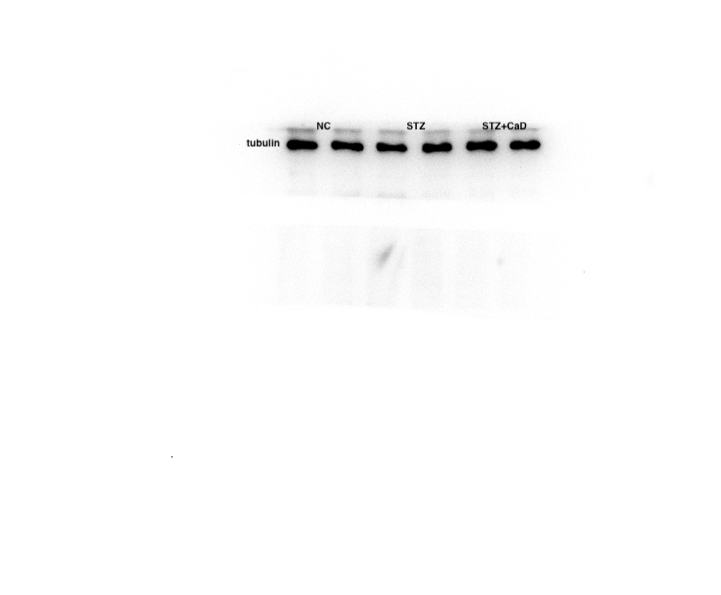

Supplement: Supplementary file 1 [file DataSheet_1.zip › original images of western blots/20.tiff]

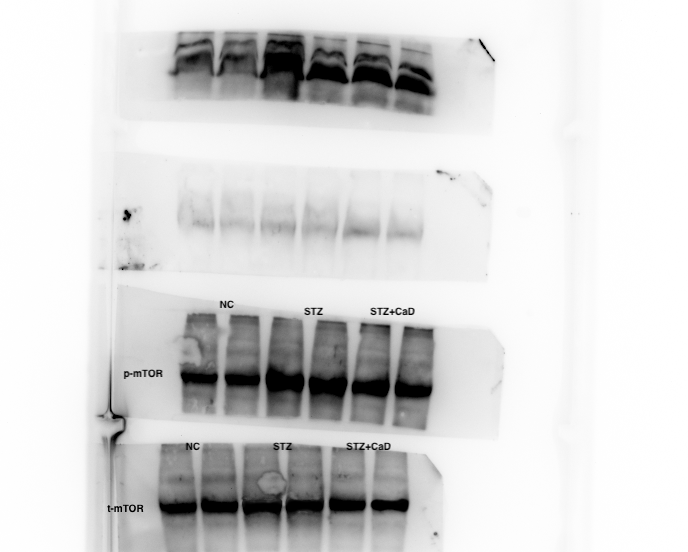

Supplement: Supplementary file 1 [file DataSheet_1.zip › original images of western blots/16.tiff]

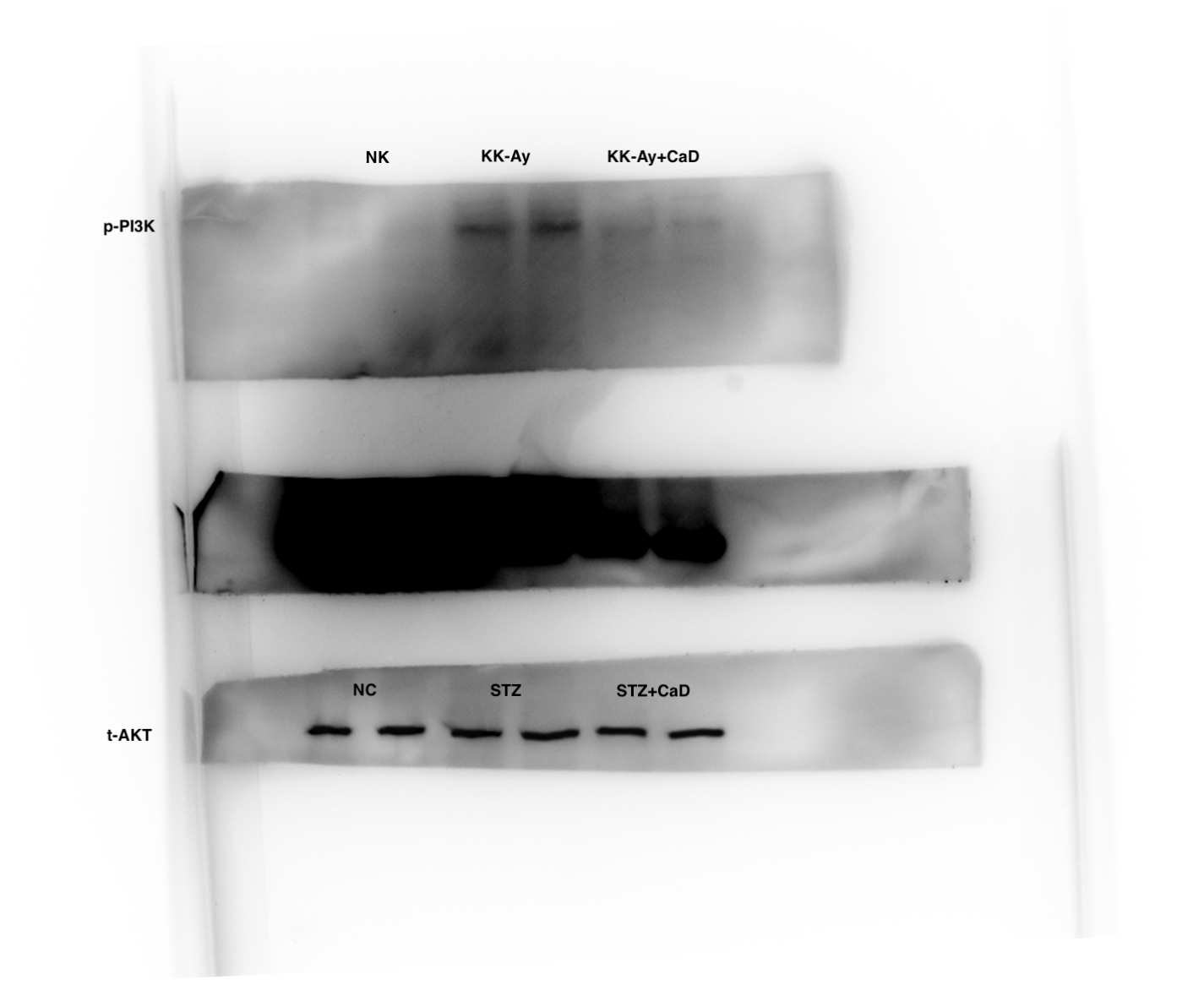

Supplement: Supplementary file 1 [file DataSheet_1.zip › original images of western blots/6.tiff]

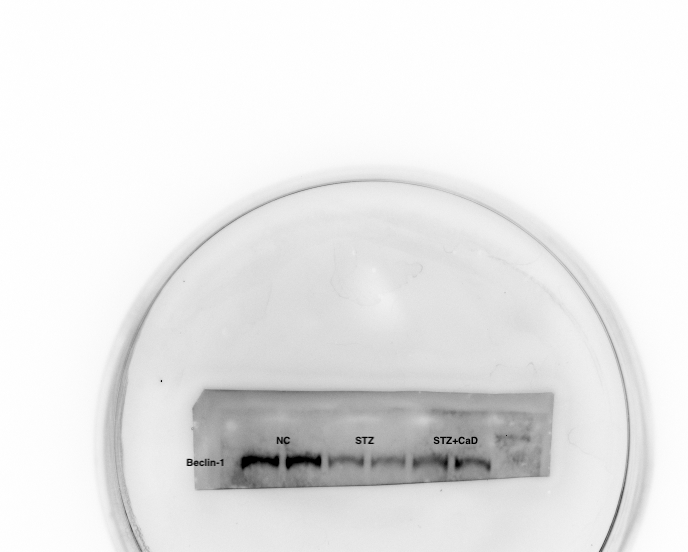

Supplement: Supplementary file 1 [file DataSheet_1.zip › original images of western blots/15.tiff]

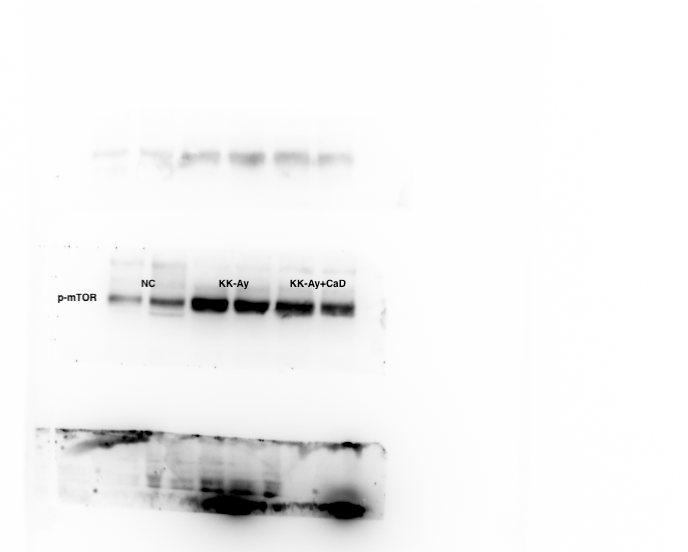

Supplement: Supplementary file 1 [file DataSheet_1.zip › original images of western blots/5.tiff]

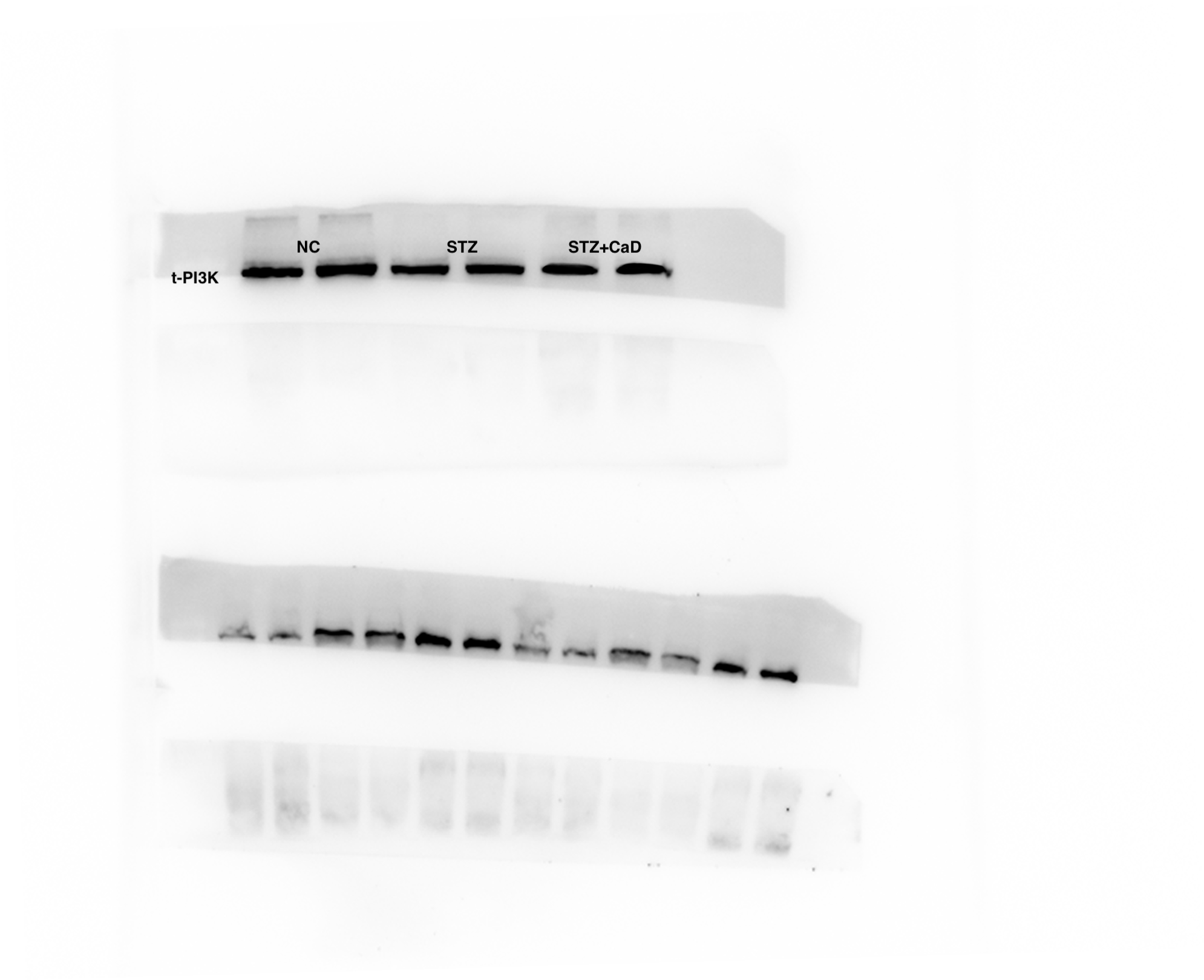

Supplement: Supplementary file 1 [file DataSheet_1.zip › original images of western blots/19.tiff]

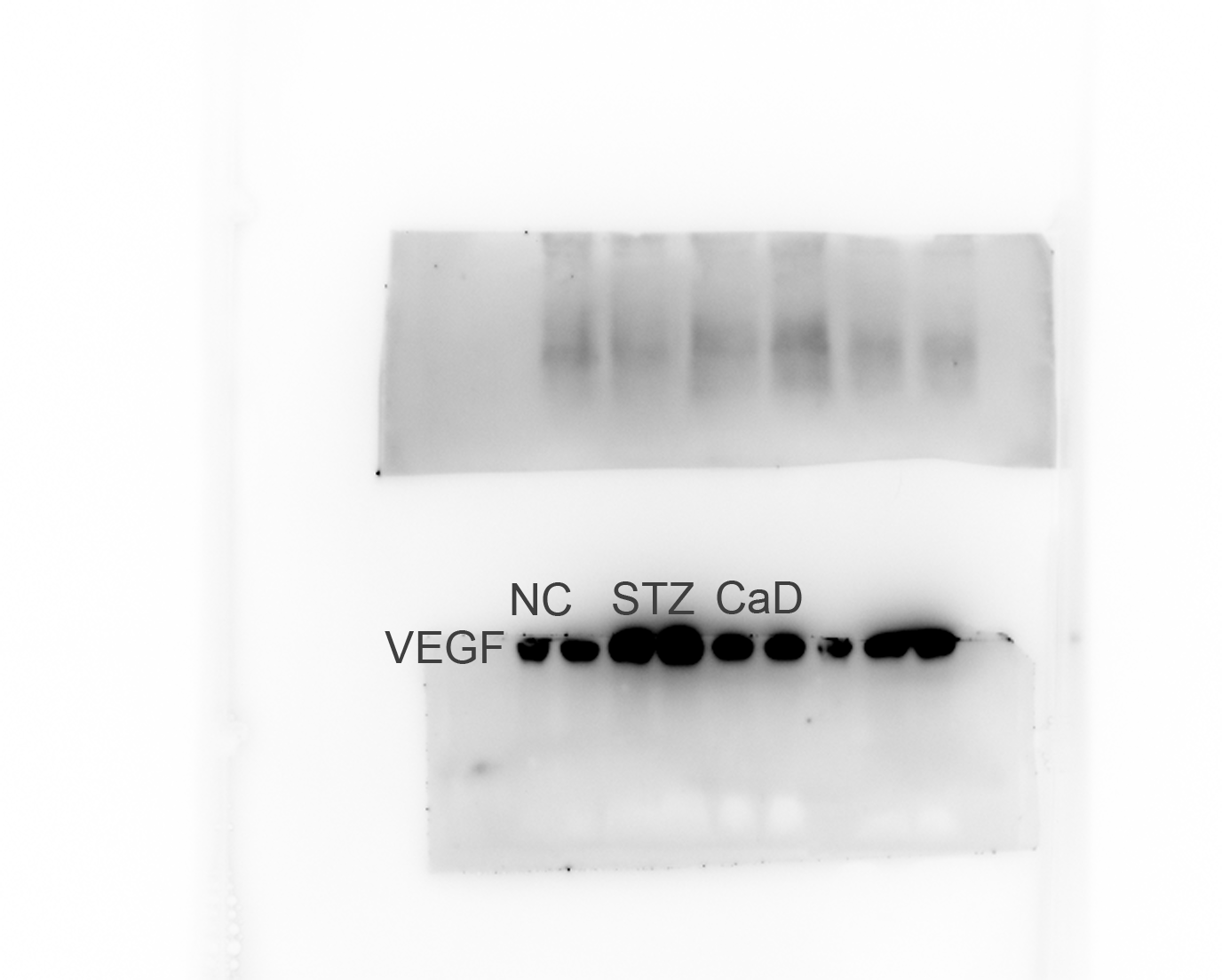

Supplement: Supplementary file 1 [file DataSheet_1.zip › original images of western blots/23.tiff]

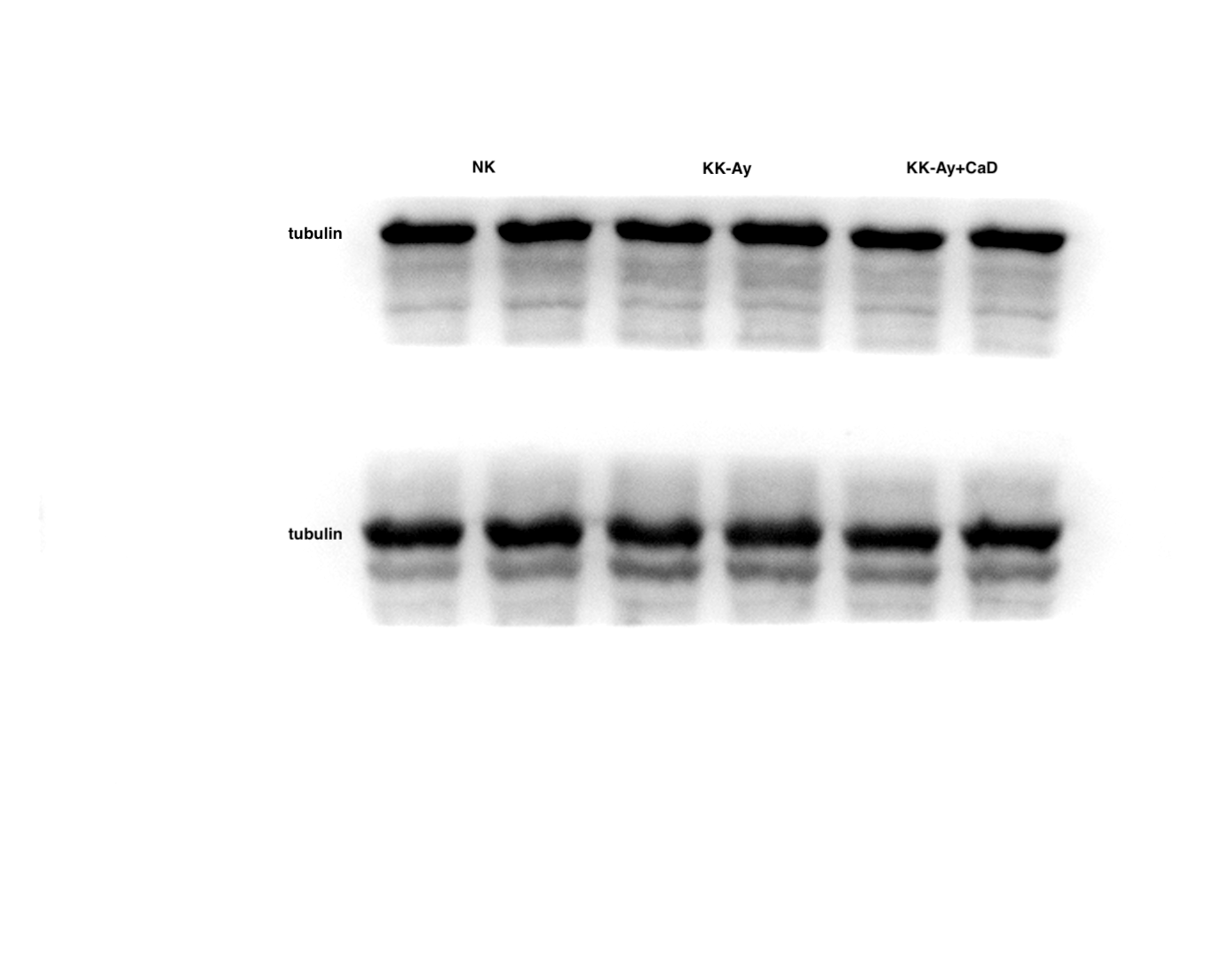

Supplement: Supplementary file 1 [file DataSheet_1.zip › original images of western blots/9.tiff]

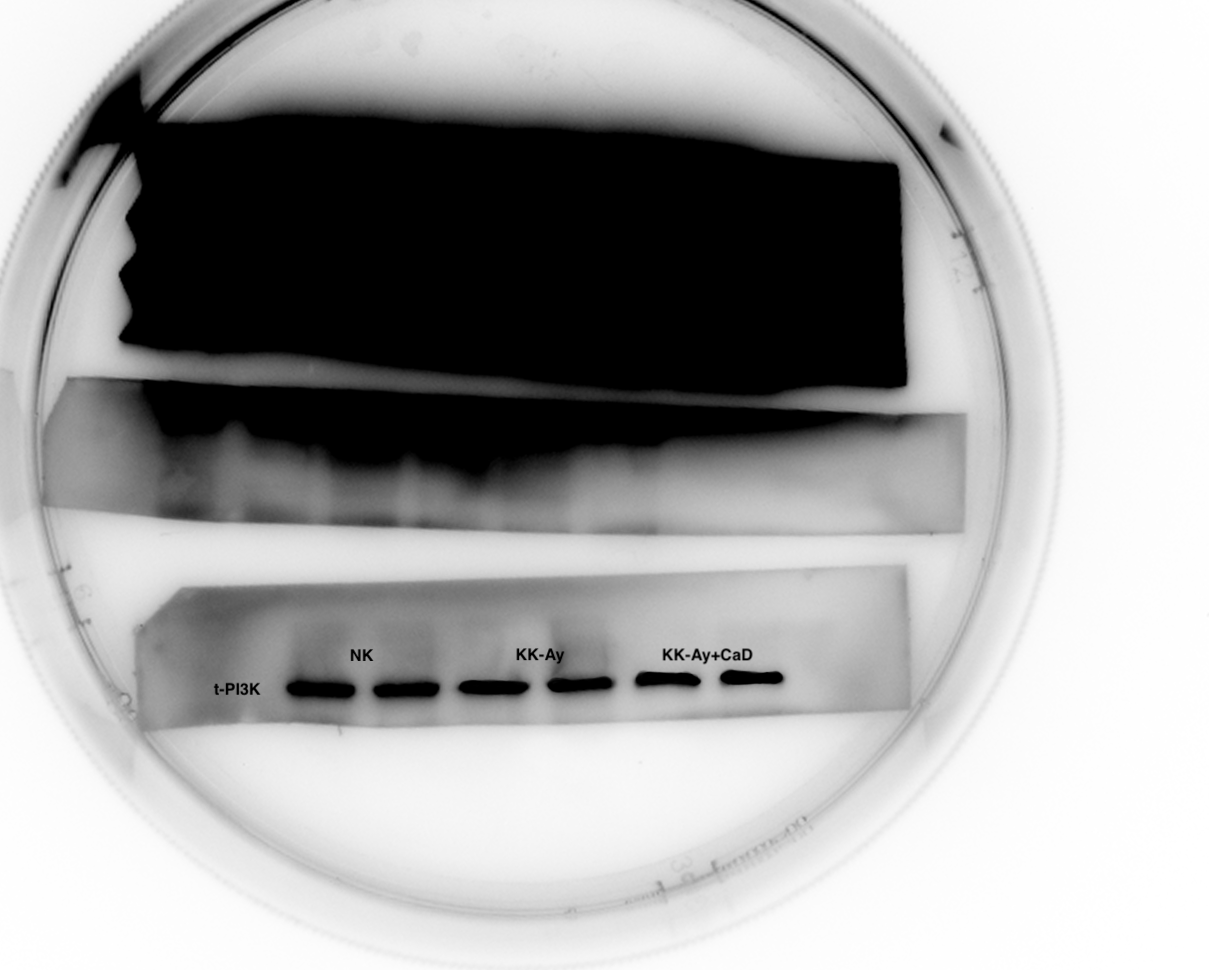

Supplement: Supplementary file 1 [file DataSheet_1.zip › original images of western blots/8.tiff]

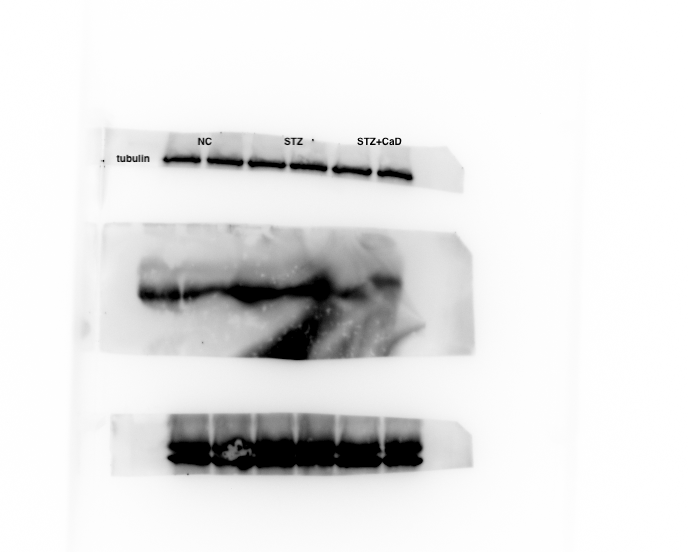

Supplement: Supplementary file 1 [file DataSheet_1.zip › original images of western blots/22.tiff]

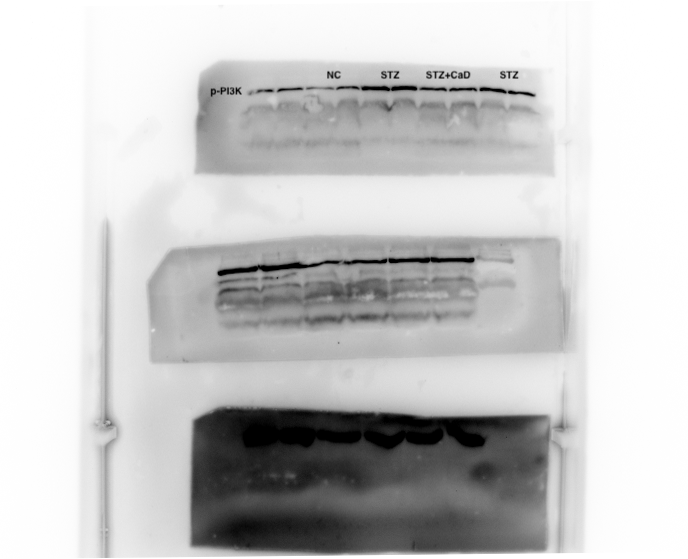

Supplement: Supplementary file 1 [file DataSheet_1.zip › original images of western blots/18.tiff]

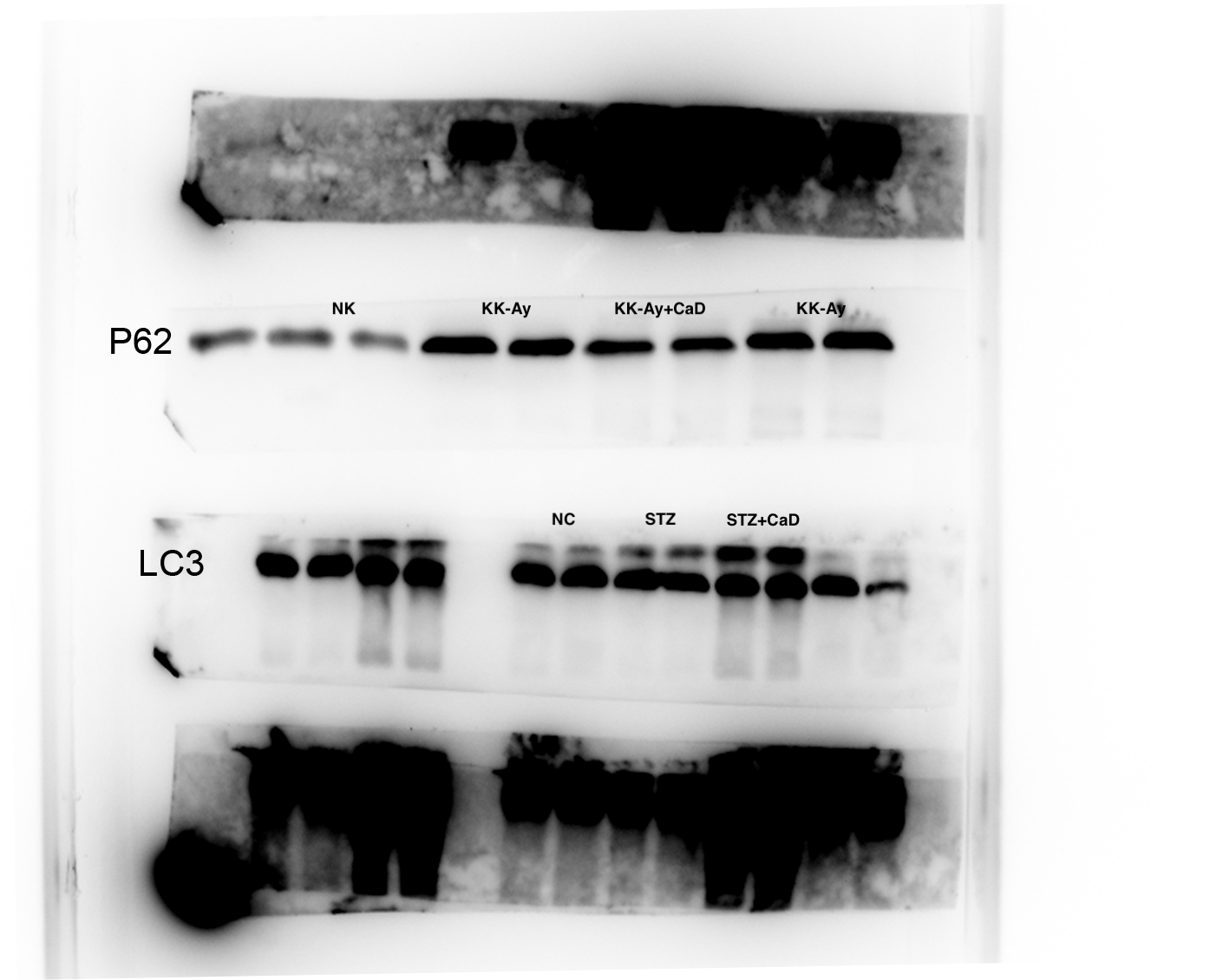

Supplement: Supplementary file 1 [file DataSheet_1.zip › original images of western blots/4.tiff]

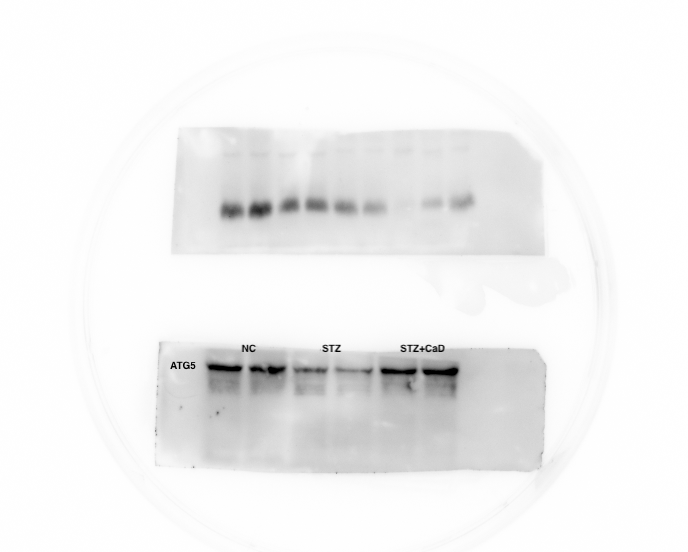

Supplement: Supplementary file 1 [file DataSheet_1.zip › original images of western blots/14.tiff]

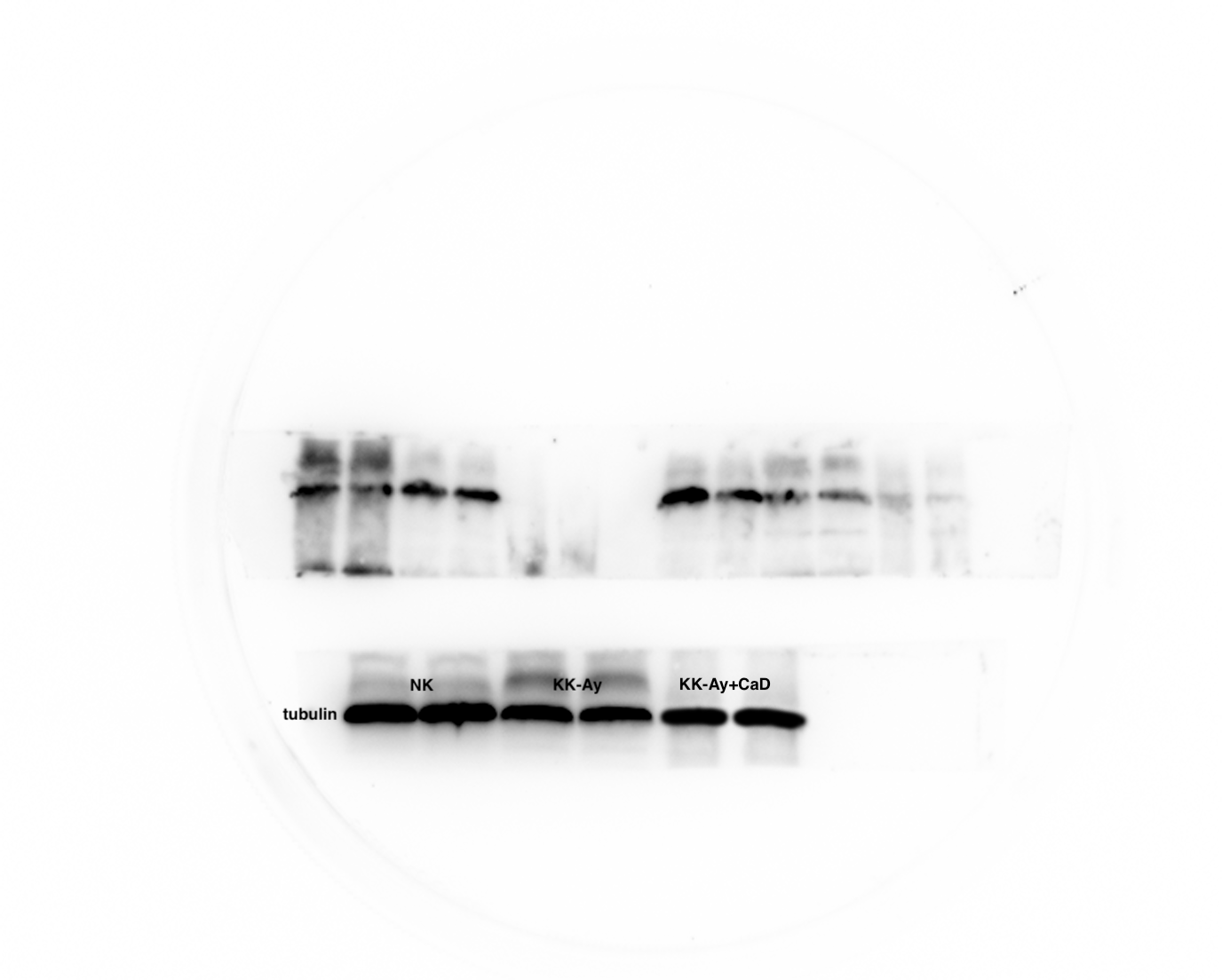

Supplement: Supplementary file 1 [file DataSheet_1.zip › original images of western blots/13.tiff]

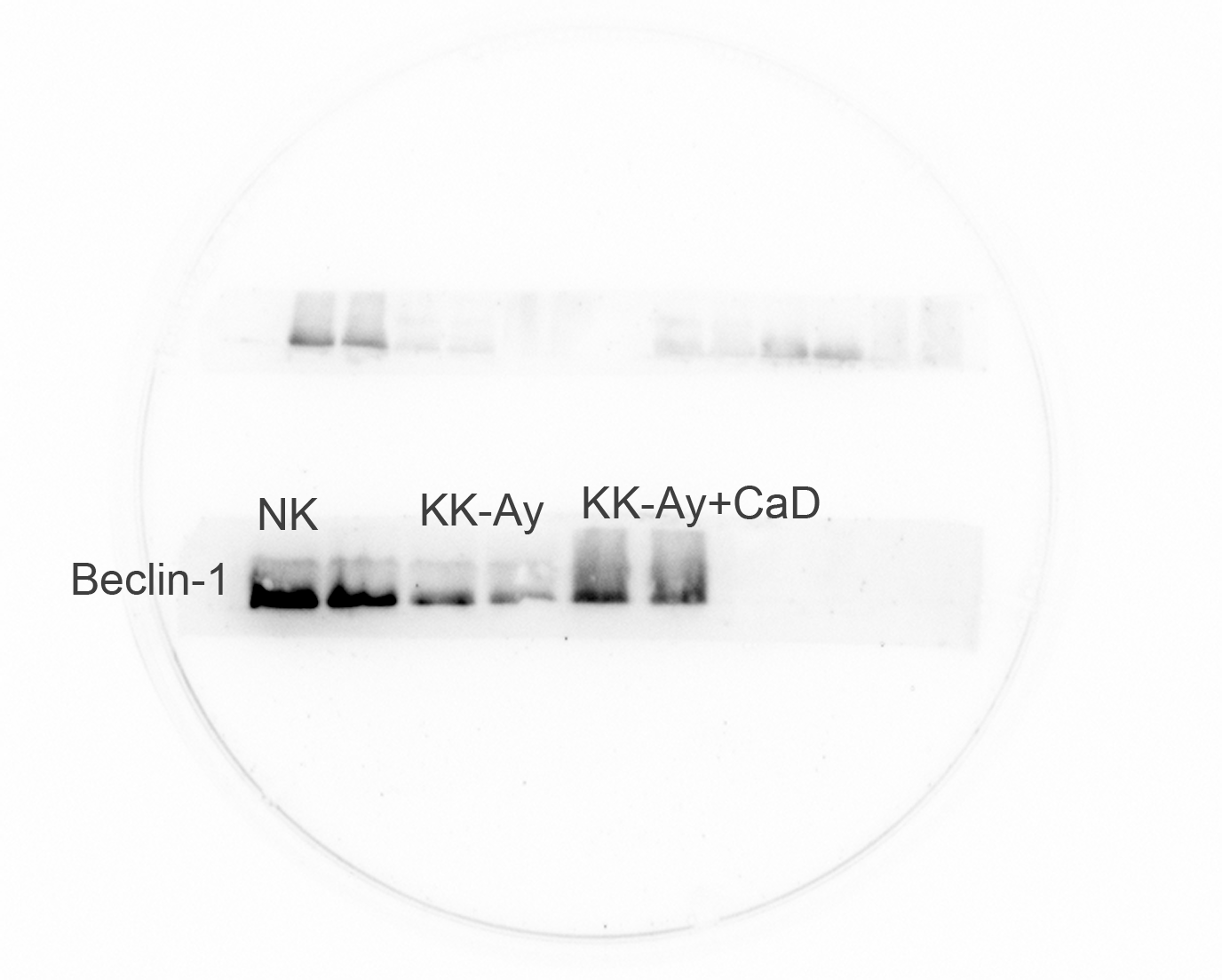

Supplement: Supplementary file 1 [file DataSheet_1.zip › original images of western blots/3.tiff]

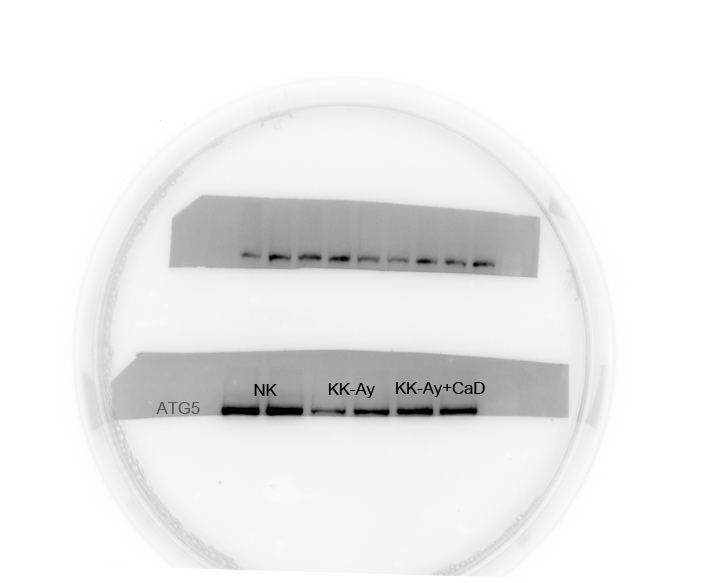

Supplement: Supplementary file 1 [file DataSheet_1.zip › original images of western blots/2.tiff]

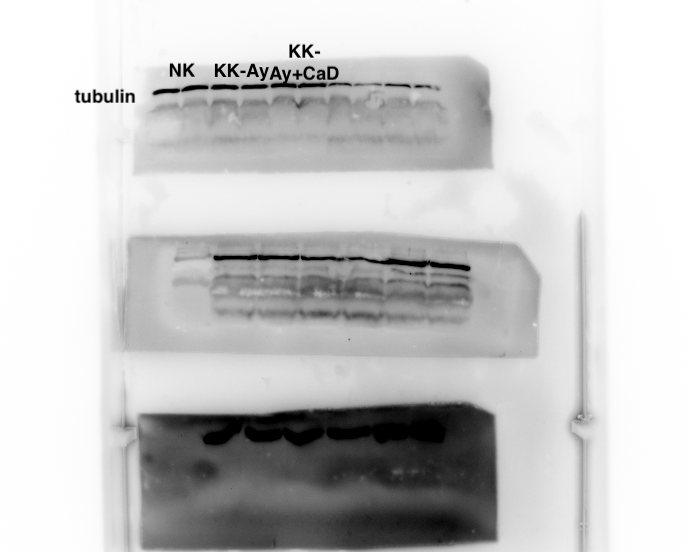

Supplement: Supplementary file 1 [file DataSheet_1.zip › original images of western blots/12.tiff]

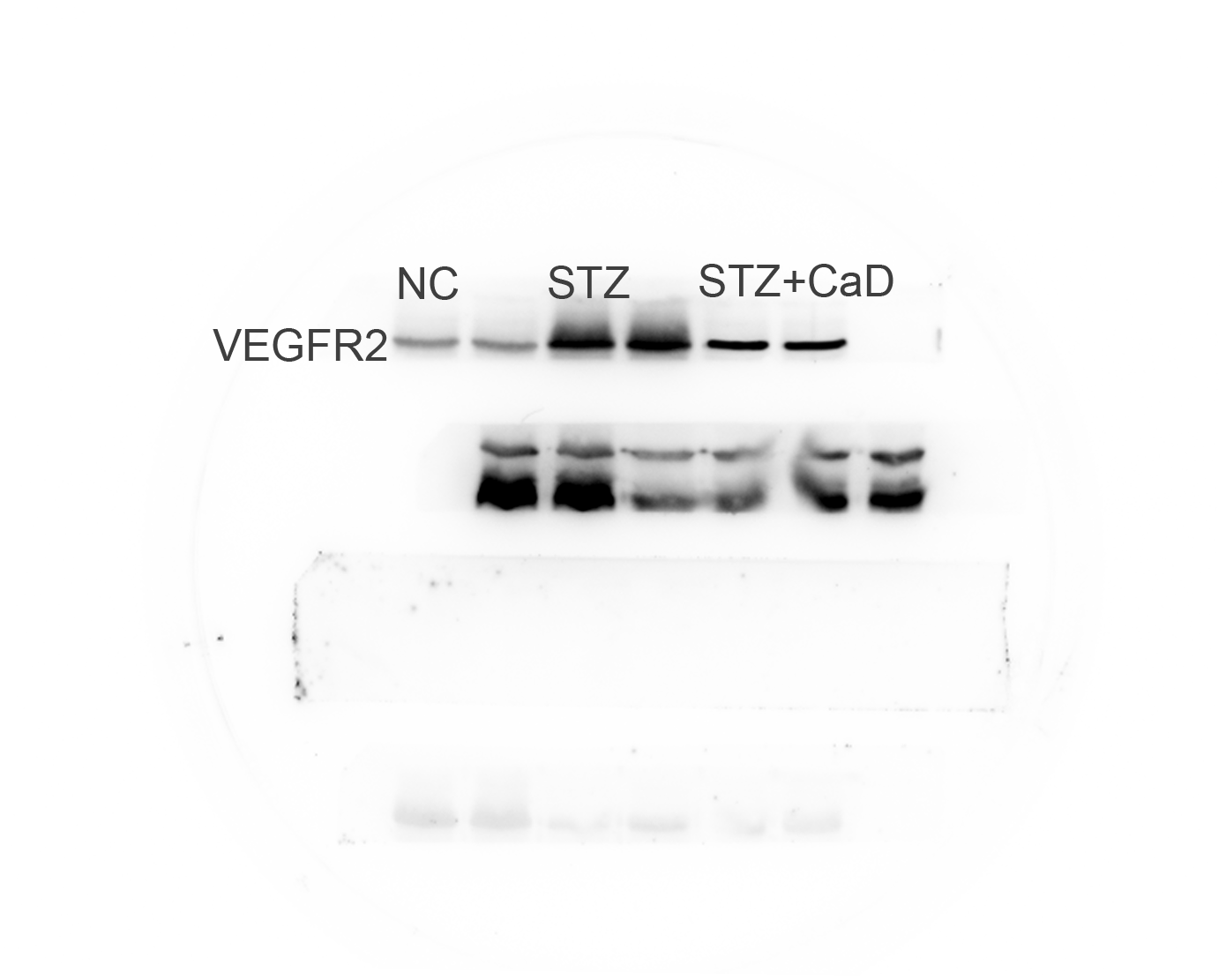

Supplement: Supplementary file 1 [file DataSheet_1.zip › original images of western blots/24.tiff]

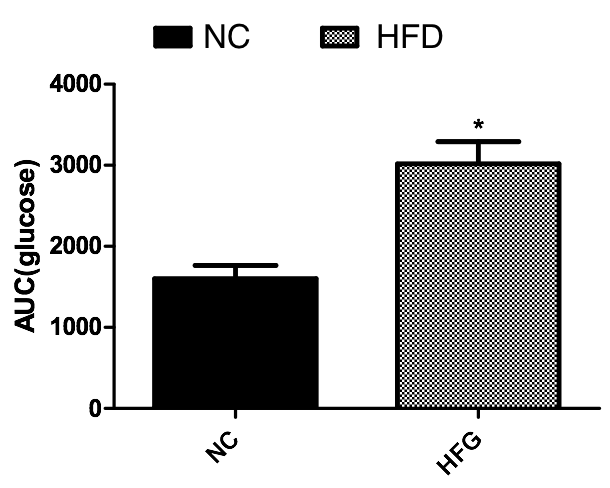

Supplement: Supplementary file 2 [file Image_1.png]

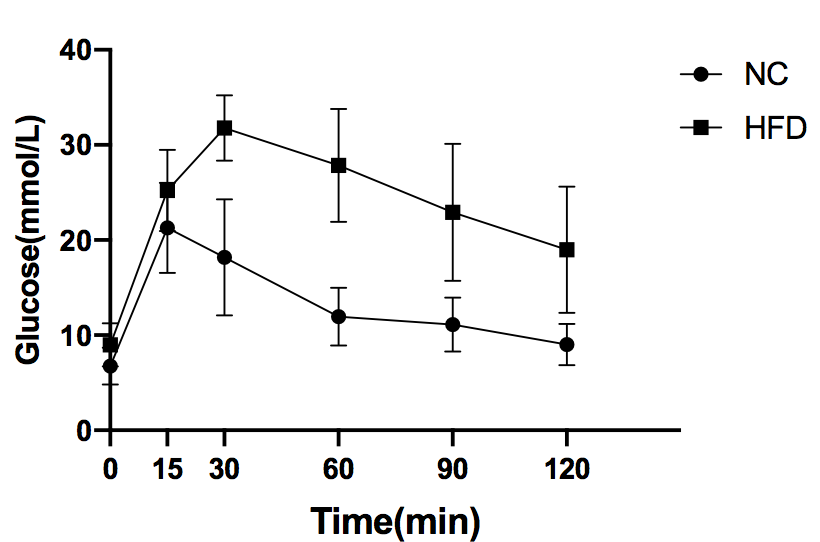

Supplement: Supplementary file 3 [file Image_2.png]

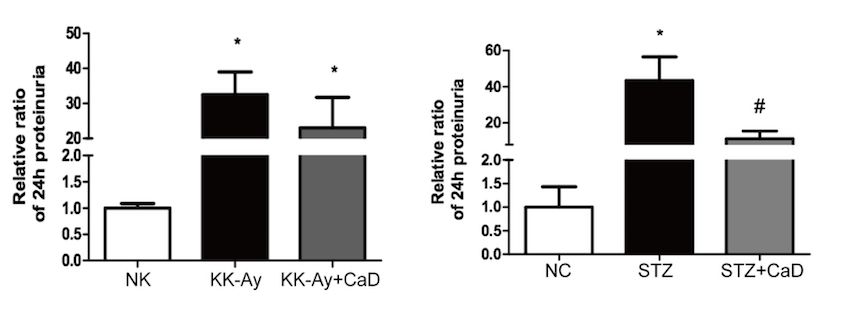

Supplement: Supplementary file 4 [file Image_3.png]

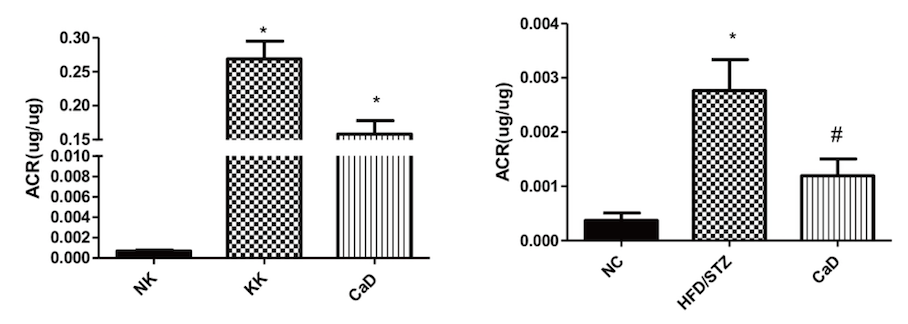

Supplement: Supplementary file 5 [file Image_4.png]

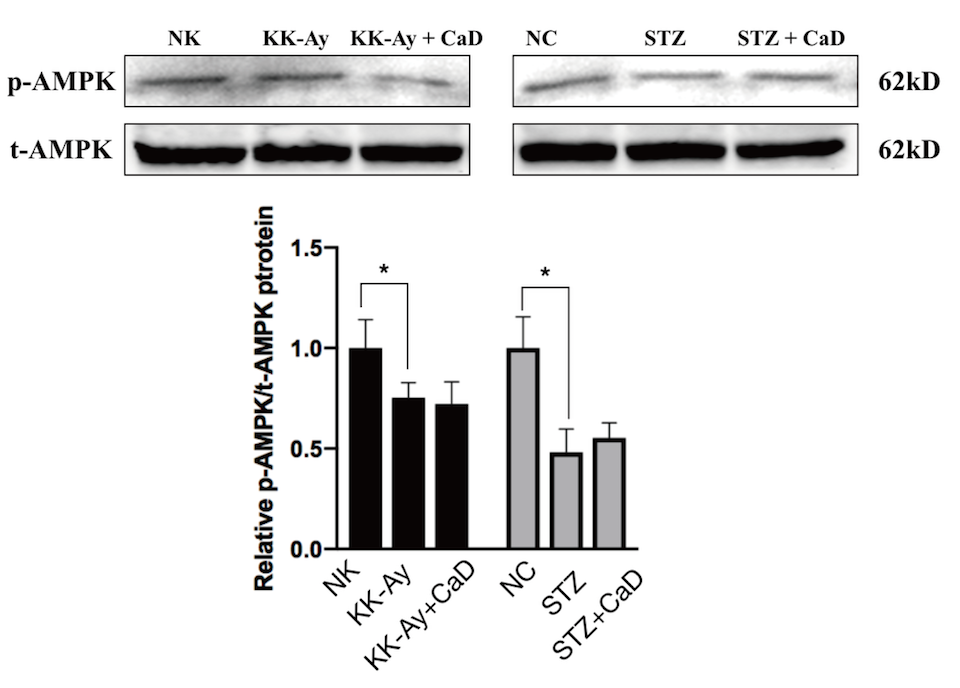

Supplement: Supplementary file 6 [file Image_5.png]
